# Almost billfish: convergent longirostry, micro‐dentition, and possible glandular sinuses in a large teleost fish from the Upper Cretaceous of Northern Italy

**DOI:** 10.1111/joa.14290

**Published:** 2025-06-25

**Authors:** Giovanni Serafini, Jürgen Kriwet, Tommaso Toldo, Eliana Fornaciari, Jacopo Amalfitano, Giorgio Carnevale

**Affiliations:** ^1^ Dipartimento di Scienze Chimiche e Geologiche Università degli Studi di Modena e Reggio Emilia Modena Italy; ^2^ Department of Palaeontology Faculty of Earth Sciences, Geography and Astronomy, University of Vienna Vienna Austria; ^3^ Dipartimento di Geoscienze Università degli Studi di Padova Padova Italy; ^4^ Dipartimento di Scienze della Terra Università degli Studi di Torino Torino Italy

**Keywords:** acrodin, comparative anatomy, Northern Apennines, oil glands, pelagic ecology, Tselfatiiformes

## Abstract

A fossilized rostrum fragment was recently reevaluated from the paleontological collections of the University of Modena and Reggio Emilia (Italy). The specimen, collected from the Northern Apennines of Modena province, was previously referred to an Eocene billfish due to the presence of cylindrical paired bones and small teeth. Thanks to nannoplankton analysis of the matrix, we reassign the specimen to the Upper Cretaceous (upper Campanian‐lower Maastrichtian). The morphological features of the rostrum, as well as its stratigraphic provenance, led us to assign the specimen to a longirostrine plethodid tselfatiiform (Actinopterygii, Teleostei) rather than to a billfish. The fragment is assumed to have originated from the mid‐posterior portion of the rostrum, with associated upper and lower jaws. The rostrum exhibits a remarkable degree of morphological convergence with extant xiphioid billfishes, together with completely unique features. The bone surface is heavily ornamented, whereas the inner structure shows a prevalence of cancellous tissue. The suture between both premaxillae and the mesethmoid is crossed by a deep longitudinal fossa not dissimilar to that of parvipelvian ichthyosaurs, a feature never reported in tselfatiiform fishes. Abundant tiny conical teeth are found between the jaw rami, separated from their sockets. Micro‐teeth can be found in both smooth‐stout form or thin and crossed by apicobasal ridges but always capped by a translucent acrodin tip. Comparative analysis with swordfish (*Xiphias gladius*) dentition provided insights on shared similarities between the two types of micro‐teeth. CT scanning of the specimen revealed a large, subtriangular, and tripartite vacuity in the upper jaw. A similar internal architecture is represented by the rostral sinus of modern billfishes, which is known to host large, globose oil‐producing glands to reduce drag on the skin. We showcase these anatomical similarities with CT scan analysis of postlarval swordfish and sailfish, together with the morphological comparison with adults of these groups from the available literature. The cumulative features gathered from the specimen suggest a fast, pelagic predatory ecology. The findings further confirm the homoplastic development of a billfish‐like body plan in Tselfatiiformes, with independently acquired morpho‐physiological adaptation that preceded the evolution of xiphioids at least since the Late Cretaceous.

## Graphical abstract

A fossil rostrum fragment of a large teleost fish from the Upper Cretaceous of Northern Italy reveals remarkable anatomical convergences with Cenozoic and Recent billfishes (marlins, swordfishes, and akin). The extinct group Plethodidae independently acquired a long snout, micro‐teeth, and oil‐gland sinuses well before the evolution of true billfishes.

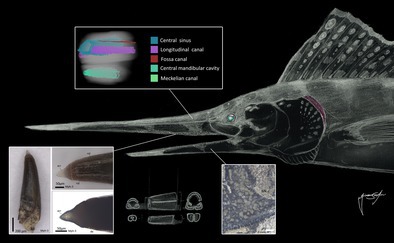

## 1 INTRODUCTION

Longirostry is a widespread cranial condition among vertebrates characterized by the anteroposterior elongation of the snout (e.g., Ballell et al., 2019; De Gracia et al., 2024; Strong et al., 2020; Wueringer et al., 2009). In longirostrine Euteleostomi (s.s. bony fishes and derived tetrapods), the condition is achieved by the allometric extension of different anterior‐most ossified elements of the skull and is often represented only by the upper jaw (Romer, 1949; Gans & Northcutt, 1983; Liem et al., 2012). Several lineages of actinopterygian fishes evolved independently a longirostrine condition in the upper jaw via three different cranial architectures:

1. Rostrum predominantly composed of elongated median, lateral, palatal (and sometimes circumorbital) paired elements of the dermatocranium (premaxillae, maxillae, nasals, vomers, palatines, etc…) as seen in the Carboniferous *Phanerorhynchus* and *Tanyrhinichthys* (e.g., Gardiner, 1967; Stack et al., 2021), in the Upper Permian to Middle Jurassic Saurichthyidae (Argyriou et al., 2018; Maxwell, 2016; Maxwell et al., 2015), in the Middle Jurassic to end‐Cretaceous Aspidorhynchidae (Brito & Ebert, 2009), in some members of the Aulopiformes (e.g., Goody, 1969), in the Eocene rhamphosid syngnathiforms (Calzoni et al., 2023), in extant and extinct Chondrostei and, most notably, in at least five Cenozoic xiphioid lineages (Hemingwayidae, Palaeorhynchidae, Blochiidae, Istiophoridae, and Xiphiidae; Fierstine, 2006; Otero, 2019; De Gracia et al., 2024). Ethmoidal derivatives can be present but are much more reduced than dermatocranial components.
2. Neurocranial rostrum (anteriorly) composed of a single elongate ethmoid derivate (i.e., mesethmoid) that anteriorly emerges and protrudes beyond the jaws, as seen in the longirostrine pachycormid *Protosphyraena* (Kanarkina et al., 2025; Woodward, 1895).
3. A rostral architecture made of a combination of Types 1 and 2, with both elongated premaxillae and an extended dorsal ethmoid component. Together with some aulopiformes (Goody, 1969), this morphotype has been described in the plethodid tselfatiiform *Rhamphoichthys* (El Hossny et al., 2023) and, to a lesser extent, in the plethodid *Martinichthys* and *Thryptodus* (Taverne, 2000a; Taverne & Gayet, 2005) up to now.

Extant billfishes (i.e., Xiphiidae [swordfishes] + Istiophoridae [sailfishes and marlins]), cumulatively referred to as “xiphioids” e.g., Fierstine, (2006) are large pelagic predators that, depending on differences in species hunting strategies, use their elongated upper jaw‐rostrum to stun, slash, and sometimes, isolate smaller prey items (fishes and squids) for subsequent whole ingestion (Domenici et al., 2014; Hansen et al., 2020; Nakamura, 1985). Additional functions of the “bill/sword” have also been suggested (e.g., hydrodynamic improvement, defense), but are considered supplementary to the prey capture purpose, behaviorally observed directly in sailfishes and generalized for other billfish species (Hansen et al., 2020). The rostrum of billfishes has a Type 1 architecture, composed of fused premaxillae overlaid dorsally by prenasal (Istiophoridae) and nasal bones, together with a lateral maxillary contribution (De Gracia et al., 2022; Fierstine & Voigt, 1996). In *Xiphias gladius*, the upper jaw elongates greatly in late postlarval stages (Govoni et al., 2003; Nakamura et al., 1951), most likely due to a negative allometry of the lower jaw rather than accelerated growth of the upper one (McGowan, 1988). In addition, members of Istiophoridae (and juveniles of Xiphiidae; Nakamura et al., 1951; Schultz, 1987; Berkovitz & Shellis, 2017) exhibit tiny teeth (sometimes referred as “micro‐teeth”) on the rostrum surface that are documented to acquit a lacerative function during prey slashing (Domenici et al., 2014; Pacher et al., 2024). Additional smaller teeth are associated with sensory dermal pits (*lacunae rostralis* sensu Häge et al., 2022). This miniaturized dentition is also reported in fossil taxa (Fierstine, 2006).

Xiphioids originated early in the Cenozoic, with the oldest fossil representatives of the group occurring from the basal Eocene strata (e.g., Monsch & Bannikov, 2012; Sytchevskaya & Prokofiev, 2002). However, at least two Cretaceous actinopterygian genera, namely the hypsocormine pachycormid *Protosphyraena* and the plethodid tselfatiiform *Rhamphoichthys*, independently acquired a bill‐shaped rostrum, medium to large sizes, and a pelagic lifestyle that likely enabled them to occupy an ecological role similar to that of billfishes (El Hossny et al., 2023). Plethodidae is an extinct family of teleost fish that inhabited the oceans from the Cenomanian to the Maastrichtian (Cooper & Norton, 2023; Shimada, 2016; Taverne & Gayet, 2005). While several plethodids are characterized by small, villiform teeth and a pointed snout (Taverne, 2000a; Taverne & Gayet, 2005), only a few genera developed a true longirostrine condition, with the most extreme representative being the genus *Rhamphoichthys* (El Hossny et al., 2023), a medium‐sized, billfish‐like taxon from the Cenomanian of Germany and Lebanon. However, the functional morphology of these Cretaceous billfish analogues is poorly understood.

Here we present the description of an incomplete rostrum from a large actinopterygian fish recovered from Campanian–Maastrichtian strata of Northern Italy. The specimen is herein tentatively referred to as a longirostrine tselfatiiform with possible affinities to the plethodids *Rhamphoichthys* and *Martinichthys*. The detailed description of the specimen, including its internal anatomy via CT‐scan, macro‐histological assessment, and microdention characterization, represents the first comprehensive analysis of the rostrum of these mysterious billfish analogues. Furthermore, thanks to a comparative analysis with modern billfishes, we provide new insights into the functional morphology of the surveyed anatomical features, deepening our understanding of the evolutionary convergence of these two distantly related pelagic teleost groups.

## 2 MATERIALS AND METHODS

### 2.1 Material

**FIGURE 1:**
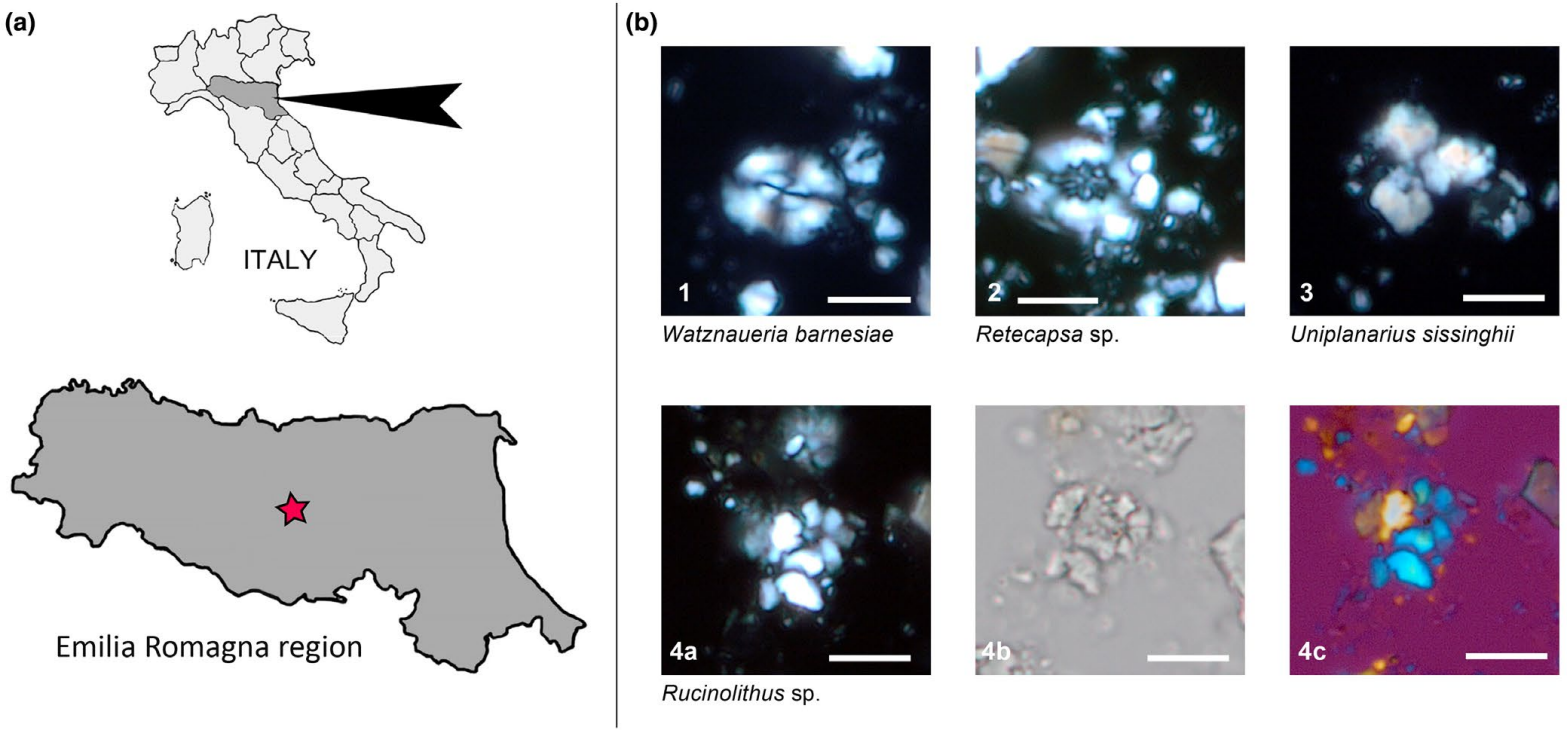
Locality map of the finding and nannotaxa associated with the matrix. (a) Pinned Emilia Romagna region in Northern Italy with star mark on the provenance of the specimen (Modena province, lower Apennines). (b) Microphotographs of calcareous nannofossils from the sedimentary matrix of IPUM 35050. *Watznaueria barnesiae*; crossed nicols. 2. *Retecapsa* sp.; crossed nicols. 3. *Uniplanarius sissinghii*; crossed nicols. 4a. *Rucinolithus* sp.; crossed nicols; 4b, same specimen; parallel light; 4c, same specimen; crossed nicols with gypsum plate. Scale bars: 5 μm.

The specimen under study (IPUM 35050) consists of the associated upper and lower jaws of a partial fish rostrum housed in the paleontological collections of the University of Modena and Reggio Emilia. The specimen lacks information on the time of acquisition, while provenance details are limited to the “lower portion” (altitude‐wise) of the Northern Apennines of Modena province (Figure 1a). Both ends had been polished, a practice that was in use in the institution from the 1990s to early 2000s for the identification of the internal structure in vertebrate fossils from the same area (e.g., ichthyosaur rostra: Sirotti & Papazzoni, 2002). Therefore, IPUM 35050 must have been part of the collection at least since the 1990s. The specimen was unofficially attributed to an Eocene billfish by Gnoli, M. in 2004–2006 (pers. comm. Serventi, P.; Vescogni, A.; Carnevale, G. 2024), but no record of any related publication was found.

### 2.2 Recent comparative material

A juvenile *Xiphias gladius* lower jaw (EMRG‐Act‐A‐82) belonging to the collection of the Evolutionary Morphology Research Group, Department of Palaeontology, University of Vienna was analyzed for comparative purposes of mandibular anatomy and micro‐dentition. CT scan data of a 33‐cm long postlarval juvenile *Xiphias gladius* wet specimen (171758) and a 21‐cm long postlarval *Istiophorus platypterus* wet specimen (187887) from the Florida Museum of Natural History were analyzed from MorphoSource to compare sinuseal osteological correlates for oil glands (UF‐Fish 171758 Head ark:/87602/m4/501620; UF‐Fish 187897 Head ark:/87602/m4/M167333, Florida Museum of Natural History (FLMNH), Division of Ichthyology, data deposited by Zach Randall. University of Florida provided access to these data, the collection of which was funded by oVert TCN. The files were downloaded from www.MorphoSource.org, Duke University). No digital derivatives (e.g., segmentations, 3D prints) were produced from these data. Images of adult *X. gladius* MRI from Videler et al. (2016) and anatomical rostral cross‐sections of adult *X. gladius* from Pazzaglia et al. (2022) were sourced from the respective papers for creating a comparative plate.

### 2.3 Osteological analysis

External morphological analysis of IPUM 35050 was carried out visually and under a Wild Heerbrugg stereomicroscope. Measurements were taken with a digital caliper at the nearest millimeter. Pictures were taken with a Canon EOS 700D with either a 50‐ or a 100‐mm lens. For osteological comparison and size estimate, IPUM 35050 was compared with the holotype of *Rhamphoichthys taxidiotis* (WMNM P 48342, LWL Museum für Naturkunde, Münster, Germany). In vivo approximate estimates of the preorbital snout length (premaxillary anterior tip–anterior margin of the orbit) and total skull length (premaxillary tip–supraoccipital) of the specimen were obtained from WMNM P 48342 scaling based on photographs and metric references from El Hossny et al. (2023). We applied two scaling measurements: (1) IPUM 35050 maximum length correlated to a 3‐cm segment between the maxilla and the beginning of the mandibular symphyseal region of WMNM P 48342; (2) upper jaw height in IPUM 35050 and WMNM P 48342 along the mandibular symphyseal area landmark (see results below). Dental plates of *Pentanogmius evolutus* (EPC 1995‐29) from the Upper Cretaceous of the Smoky Hills Chalk (Kansas, US) were figured in a comparative plate together with WMNM P 48342.

### 2.4 Dental analysis

Nine teeth of IPUM 35050 were mechanically extracted with a hypodermic needle and cleaned from the encrusting matrix with 1% formic acid. Four teeth were photographed with a Keyence VHX‐X1 digital microscope in both direct and transmitted light. The remaining extracted micro‐teeth were carbon‐coated and analyzed with a Jeol JSM‐6010 plus/LA SEM. An isolated plethodid tooth (VP‐15224, Sternberg Museum) from the Upper Cretaceous of the Greenhorn–Lincoln formation (Colorado, US) was used as a comparison for the presence of apical acrodin.

### 2.5 CT scan

To evaluate the internal structure of IPUM 35050, the specimen was CT scanned with a NSI x5000 at TEC Eurolab (Campogalliano, Modena). A first binning scan was conducted with a voxel resolution of 135 μm, 240 kV voltage, 540 μA current, and a focal spot size of 145 μm. A second flat panel scan was conducted with a voxel resolution of 70 μm, 240 kV voltage, 600 μA current, and a focal spot size of 145 μm. Both scans used a 2520DX Varian detector. Slices were processed with the Dragonfly Package Software (v. 2022).

### 2.6 Nannoplankton analysis

Three samples of matrix were extracted with either a Dremel or a scalpel after HCl immersion of the tip/blade to avoid calcareous contamination. To analyze the calcareous nannofossil content, samples were processed according to the smearing technique (Bown & Young, 1998). Analysis was conducted under a polarized light microscope with a magnification of 1250X.

### 2.7 Compositional analysis

The skeletal tissue of IPUM 35050 was analyzed with a transportable XRF (Bruker Artax MNU‐3 with helium‐controlled atmosphere) to test its surface composition without damaging the specimen. Relative spectra were processed with Artax software.

## 3 PALEOENVIRONMENTAL SETTING

The specimen under study originates from the lower part of the Northern Apennine extending south of Modena province (Emilia Romagna region, Northern Italy; Figure 1a). The area is geologically very heterogeneous, with both Mesozoic and Cenozoic units representing marine deposits. Cretaceous units of the area belong to the Ligurian Complex, deep‐sea sediments deposited in what is now the Ligurian Sea and brought north‐eastward during the Apennines orogenesis (Bettelli et al., 1989, 2002). Most of these units are lithologically highly heterogeneous but can be identified by the shared presence of multicolored pelagic clays, together with calcilutitic, siliceous, and arenitic turbidites (Bettelli et al., 2002). Due to the complex tectonic history of the Apennine chain, these units no longer (or extremely rarely) outcrop in their original stratigraphic succession (Bettelli et al., 2002). Moreover, as these units were deposited below the carbon compensation depth (CCD), the biostratigraphy of the complex by means of calcareous nannofossils is commonly unsuccessful. This complex is believed to be representative of an abyssal plain palaeoenvironment deposited in the Ligurian–Piedmont Ocean in the westernmost side of the Tethys (Bettelli et al., 2002; Serafini et al., 2023). This paleoenvironmental interpretation is supported by the extreme rarity of calcitic micro‐macrofossils (being dissolved under the CCD), together with the very rare occurrence of continental materials and by the distinctive presence of polymetallic nodules/ferromanganese crusts (both typical of present‐day abyssal plains, e.g., Blöthe et al., 2015; Kuhn et al., 2017) associated with seabed sediments, ichnofossils, and vertebrate carcasses (Serafini et al., 2023). Additionally, only pelagic vertebrate remains from these Cretaceous units have been reported, among which lamniform sharks (Serafini et al., 2023), platypteriigine ichthyosaurs (Serafini et al., 2022), and mosasaurine mosasaurs dominate (Fanti et al., 2014; Palci et al., 2014).

## 4 RESULTS

### 4.1 Age determination

The calcareous nannofossil content from the sampled matrix of IPUM 35050 is exceedingly scarce and poorly preserved. Nevertheless, all the specimens identified can be confidently attributed to the Cretaceous. In particular, the following species were identified: a few specimens of *Watznaueria barnesiae*; a single specimen of *Retecapsa*; a single specimen of *Rucinolithus*, possibly pertaining to *Rucinolithus hayi*, a species that is restricted to the Campanian stage; and a single specimen of *Uniplanarius sissinghii* (Figure 1b). The main diagnostic features of *Uniplanarius sissinghii* are the four narrow, relatively long, ray‐like elements, which sometimes exhibit a small central quadrate diaphragm. The specimens illustrated (Figure 1b_3_) lack one of the four long, tapering rays; however, the sutures remain clearly visible. The presence of *U. sissinghii* indicates that the sample belongs to zones CC21–uppermost CC23 of Sissingh (1977) or to UC15c–uppermost UC17 of Burnett (1998) biozonation. Consequently, the age of the sample IPUM UN1 spans from the upper middle Campanian to the lower early Maastrichtian.

### 4.2 Taphonomy and preservation

**FIGURE 2:**
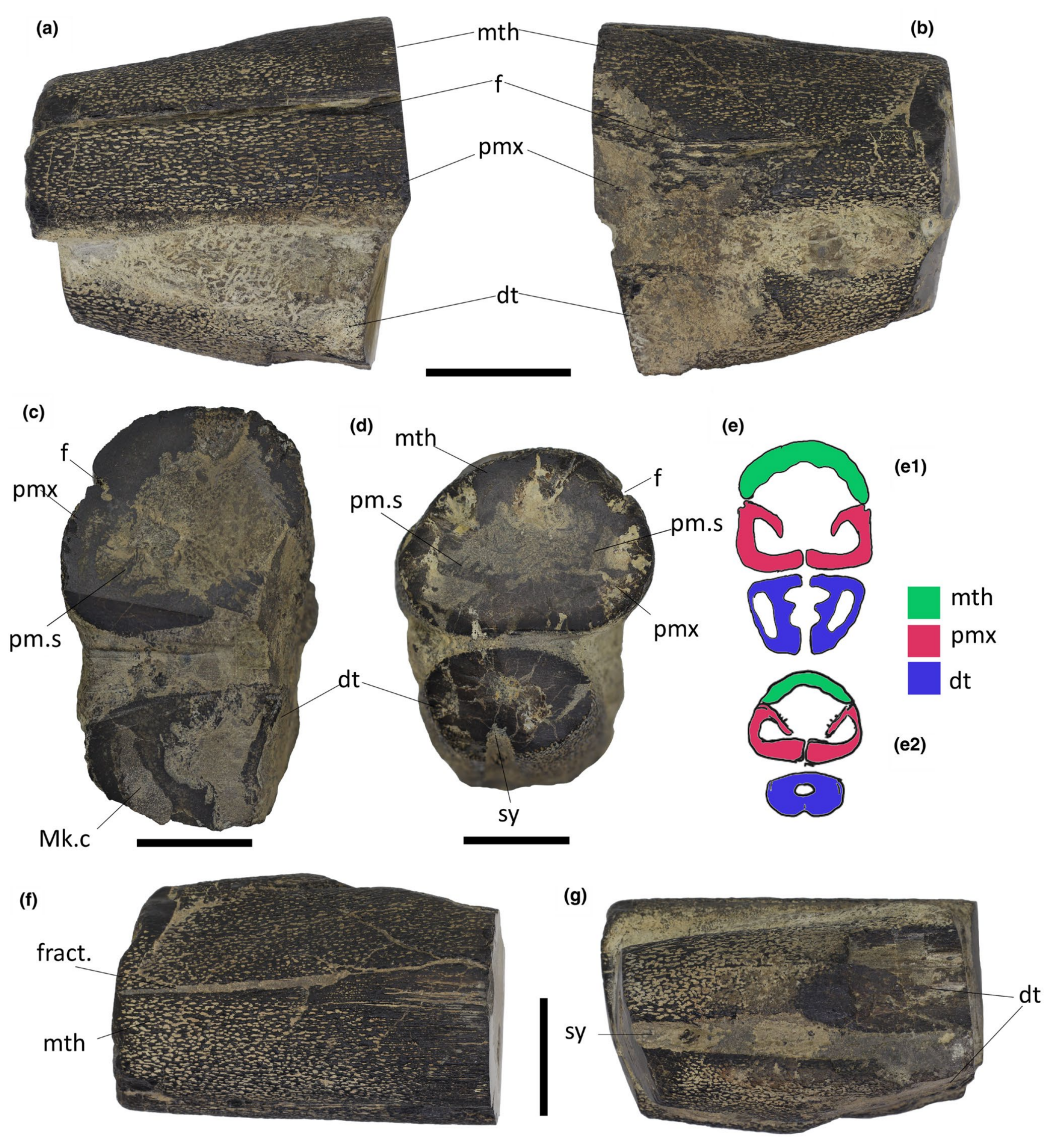
Overview of IPUM 35050. (a) Left‐hand side of the specimen. (b) Right‐hand side of the specimen. (c) Posterior view of the sectioned and polished end of the specimen. (d) Anterior view of the sectioned and polished end of the specimen. (e) Interpretative anatomical drawing of the posterior (e_1_) and anterior (e_2_) rostrum fragment without any deformation. (f) Dorsal view of the specimen. (g) Ventral view of the specimen. Dt, dentary; Mk.c, Meckelian canal; mth, mesethmoid; f, fossa; fract., fracture; pm.s, premaxillary septum; pmx, premaxilla; sy, symphysis. Scale bars: (a, b) 5 cm; (c, d, f, g) 3 cm.

**FIGURE 3:**
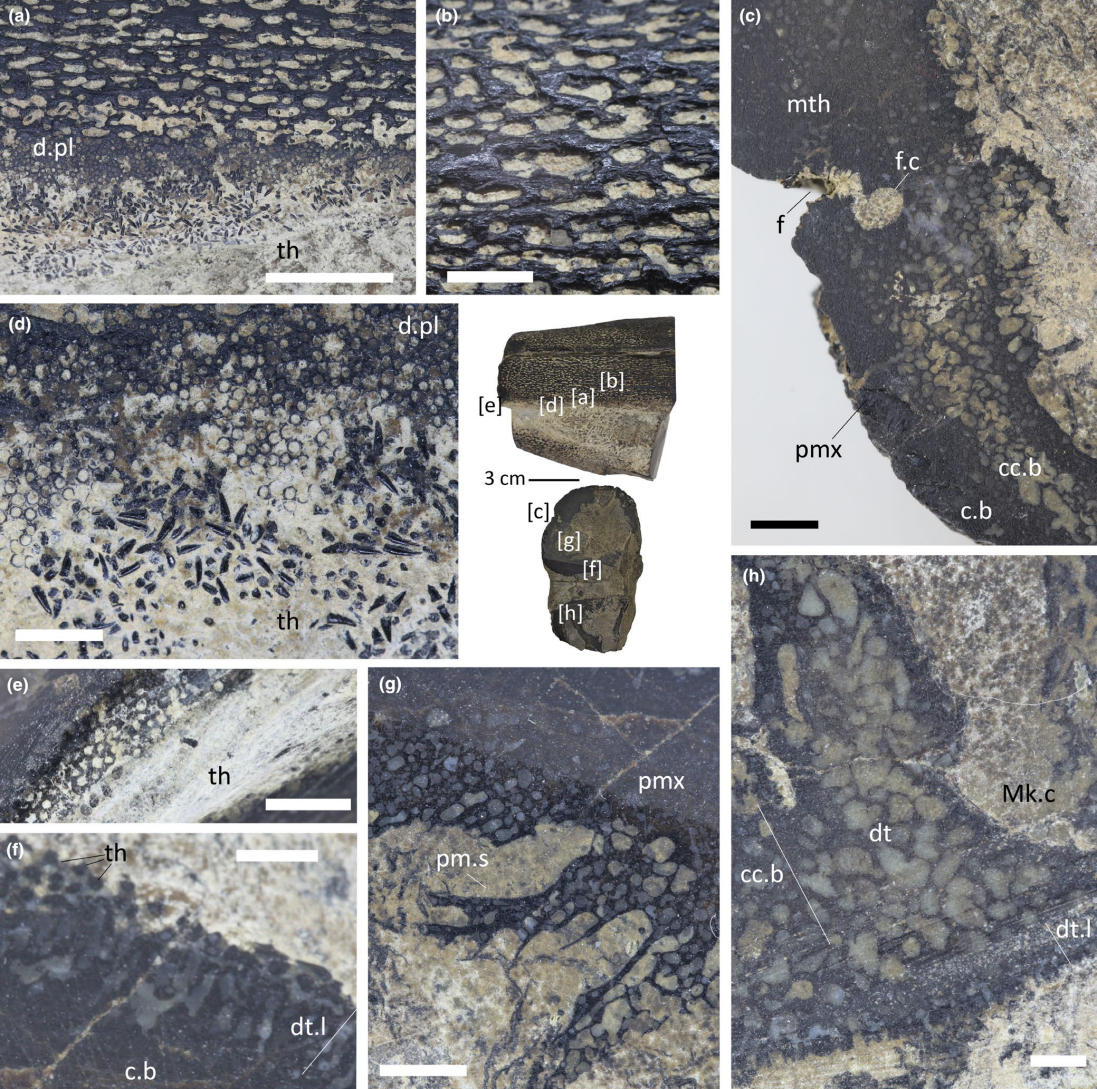
Details of IPUM 35050. (a) Premaxilla ornamentation and dental plate. (b) Close‐up of mesethmoidal ornamentation. (c) Detail of the mesethmoid‐premaxilla contact and fossa in cross section. (d) Pitted alveoli and dislodged micro‐teeth under the premaxilla. (e) Alveoli in cross section at the floor of the premaxilla. (f) Sectioned dentigerous layer near the premaxillary symphysis. (g) Sectioned premaxillary septum with detail of its cancellous histology. (h) Sectioned dentary ramus in proximity to the Meckelian canal with detail of its cancellous histology. Position of each panel is reported in brackets at the center of the picture. c.b., compact bone; cc.b, cancellous bone; d.pl., dental plate; dt.l, dentigerous layer; Mk.c, Meckelian canal; pm.s, premaxillary septum; pmx, premaxilla; th, tooth. Scale bars: (a) 1 cm; (b, f, h) 2 mm; (c, d, e, g) 5 mm.

IPUM 35050 is preserved three‐dimensionally with almost no sign of deformation (Figure 2). Skeletal and dental tissues appear completely non‐eroded, with only two portions of abraded bone in the posterior right premaxilla and dentary attributable to recent weathering. As jaw rami are found still associated, the carcass must have experienced little disturbance at the seafloor; as such, the fragmentary nature of IPUM 35050 is likely caused by post‐diagenetic tectonic activity. IPUM 35050 is histologically coherent and preserves exquisite details of its original skeletal and dental organization (Figure 3). Compositional TXRF analysis revealed the widespread presence of manganese and iron on both the cortical and cancellous bone of IPUM 35050 (S1). CT scan analysis corroborates the integrative presence of these metals in the bones, as the specimen is structurally extremely dense (Hounsfield values over 3000 HU) and barely permeable by x‐rays. Comparative taphonomy with other marine vertebrate remains from the Ligurian Complex (Serafini et al., 2022, 2023; Serafini et al. preliminary observation) suggests that similar environmentally driven biostratinomic features and diagenesis might have led to the deposition and permeation of metallic compounds on IPUM 35050. The polymetallic alteration of the specimen supports the hypothesis that the specimen was deposited in an abyssal plain paleobiotope (Blöthe et al., 2015; Serafini et al., 2023).

### 4.3 Description

IPUM 35050 is composed of two cylindrical associated jaw rami, held in anatomical position by the encrusting matrix (Figure 2a–d). The specimen reaches 11.5 cm in length and 11 cm at its maximum height (posterior end). Both the anterior and posterior ends of the upper and lower jaw have been polished, revealing the internal structure (Figure 2c–e). The skeletal tissue is black in coloration and particularly dense (Figure 3a–f); however, the sectioned bone reveals the widespread presence of alveolated cancellous bone already a few millimeters beneath the surface (Figure 3c,g,h); as highlighted by the compositional analysis (see discussion above), the high density of the bones is caused by metallic permeation of the skeletal tissue rather than intrinsic histological features. Both jaws bear dental plates with hundreds of millimetric pitted sockets (Figure 3a,d–f); closely associated with these sockets (or spread on the encrusting matrix), tens of millimetric conical teeth can be observed mostly on the left side of the rostrum (Figures 2a,b and 3a,d). Only a few teeth are in fact recognizable on the right side, possibly being obscured by the encrusting matrix or perhaps having dethatched from the rostrum side facing worse preservation conditions during carcass settling on the seafloor. Bones and dentition are singularly described below in dedicated paragraphs.

#### 4.3.1 Mesethmoid

**FIGURE 4:**
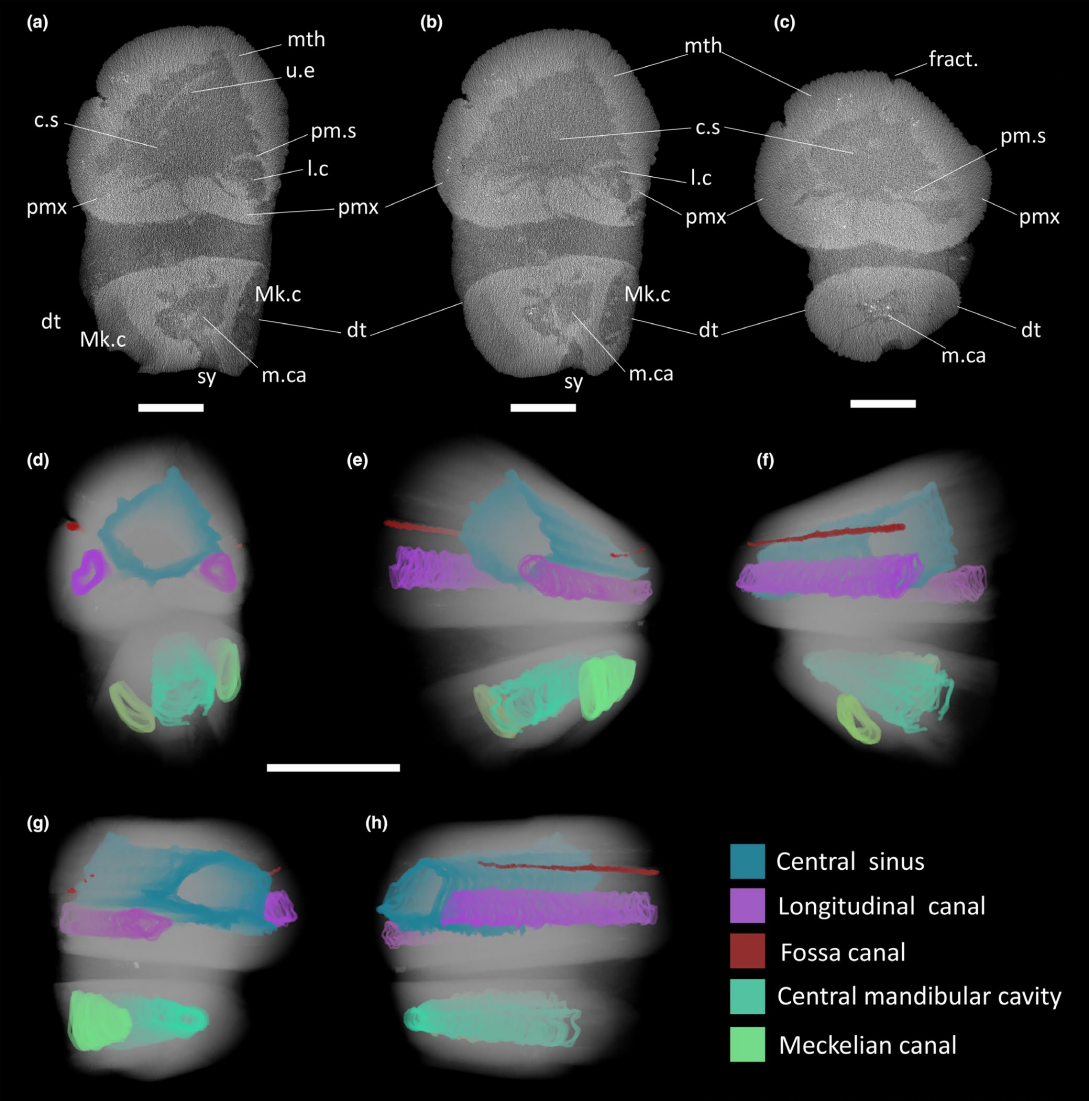
CT‐scan of IPUM 35050. (a) Posterior axial slice of the specimen. (b) Median axial slice of the specimen. (c) Anterior axial slice of the specimen. (d–h) Volumetric rendition of the scan with segmented cavities. c.s, central sinus; dt, dentary; fract., fracture; l.c, longitudinal canal; m.ca, central mandibular cavity; Mk.c, Meckelian canal; mth, mesethmoid; pm.s, premaxillary septum; pmx, premaxilla; u.e, unidentified element. Scale bars: (a‐c) 2 cm; (d‐h) 5 cm.

The first bone that can be identified antero‐posteriorly is a partial mesethmoid running dorsally throughout the entire specimen length, forming the roof of the rostrum (Figure 2a–f). The identity of this bone was determined due to its solid and unpaired appearance; a longitudinal furrow that unevenly crosses the surface of this element (Figure 2f) is recognized as a crack and not as a medial symphysis, a feature also supported by axial CT slices (Figure 4a–c). The preserved mesethmoid is dorsoventrally arched and semicircular in cross section but gradually flattens towards the anterior end. The surface of the mesethmoid is initially (posterior‐most) characterized by an ornamentation made of densely packed longitudinal ridges but quickly transits to a well‐defined reticular ornamentation that persists till the anterior end. The anastomosed net‐like ornamentation is seemingly achieved by smaller transverse projections of the longitudinal ridges that contact each other. Being these ridges and “ridgelets” slightly elevated, they form irregularly elongated (subrectangular) pits that were infilled by matrix (Figure 3b). The ornamented dermal component (sensu Jollie, 1986) is well developed and dome‐shaped in cross section. The CT scan analysis exposes the internal organization of the mesethmoid: the floor of the bone appears irregular, with at least three longitudinal grooves that run antero‐posteriorly (Figure 4a,b). Due to the high structural density of the specimen, it is unclear if these grooves are vascularly connected with the roof of the bone.

#### 4.3.2 Premaxillae

Two rounded rami neighbor the mesethmoid, here interpreted as portions of the paired premaxillae (Figure 2a–e). The reticular ornamentation described above persists in these elements. The left ramus is the best representative of the original anatomy, being the right one posteriorly abraded and missing a small portion (Figure 2b). The two rami are internally curved, making both the lateral walls and the floor of the preserved upper jaw. The ventrally conjoined premaxillary rami therefore create a false palate (Figures 2c,d and 4a–c). The dorsal portion of the premaxillary rami also bent downwards, separating sections of the central rostral vacuity (see description below) with two septa. As for the mesethmoid, the premaxillae anteriorly tapered, resulting in the slight narrowing of the rostrum fragment. The most peculiar feature of these elements is their contact with the mesethmoid: on both sides (but better appreciable on the left one), the suture between the two bones coincides with a distinctive fossa that runs longitudinally across the entire specimen length (Figure 2a). This fossa is relatively deep (5 mm) and in cross section, as judged from the polished posterior end, appears to be infolding on itself (Figure 3c), contacting a neighboring canal that runs longitudinally inside the premaxilla (Figure 4d–h). Due to the limited x‐ray permeability of the bones, it is unclear, based on the CT data, whether and where this canal branches internally. Both premaxillary rami bear numerous tooth sockets (and their relative displaced small teeth). These sockets drape the lower portion of both lateral parts of the premaxillae (Figure 3a,d); thanks to the cross section, these tooth‐bearing areas can be observed persisting internally, covering the entire premaxillary floor till the symphysis (Figure 3e,f).

#### 4.3.3 Unidentified internal upper jaw element

In the posterior‐most axial CT slices of the specimen, a plate‐like bone fragment can be observed persisting inside the internal vacuity for some centimeters (Figure 4a). Currently, we are unable to confidently identify this element, possibly representing another cranial component, a displaced fragment of the vomer, or perhaps one of the nasals.

#### 4.3.4 Dentary

The preserved lower jaw of IPUM 35050 is composed of a single paired bone, here identified as the dentary (Figure 2a–d,f). These bones are affected by surface abrasion that exposed their internal structures and obscured details of their overall morphology. Although damaged and incomplete, the posterior end is higher than the anterior one, suggesting the same narrowing trend as seen in the upper jaw. Posteriorly, the two dentary rami exhibit their Meckelian canals in cross section (Figure 2c), which are well developed at their posterior end (2.9 cm high) but rapidly reduce and seemingly close towards their anterior end. The ventral symphysis of the dentary is deep and wide, as the two rami strongly infold internally. The dorsal symphysis between the two rami is instead extremely narrow and visible only in posterior‐most sections of the CT scan (Figures 2d and 4a–c). Anteriorly, the dorsal symphysis disappears, as the two rami completely fuse forming the mandibular roof. This arrangement creates a central cavity that posteriorly merges with the symphysis, while it gradually separates into an enclosed and smaller central channel towards the anterior end (Figure 4). The same reticular ornamentation of the upper jaw elements is also developed on the dentaries. The left dentary has numerous displaced tiny teeth on its anterior lateral side, but sockets are free of matrix only on a small tooth‐bearing patch on the right ramus. As for the premaxillae, these toothed areas extend also internally, on the dentary roof (Figure 3h).

#### 4.3.5 Dentition

**FIGURE 5:**
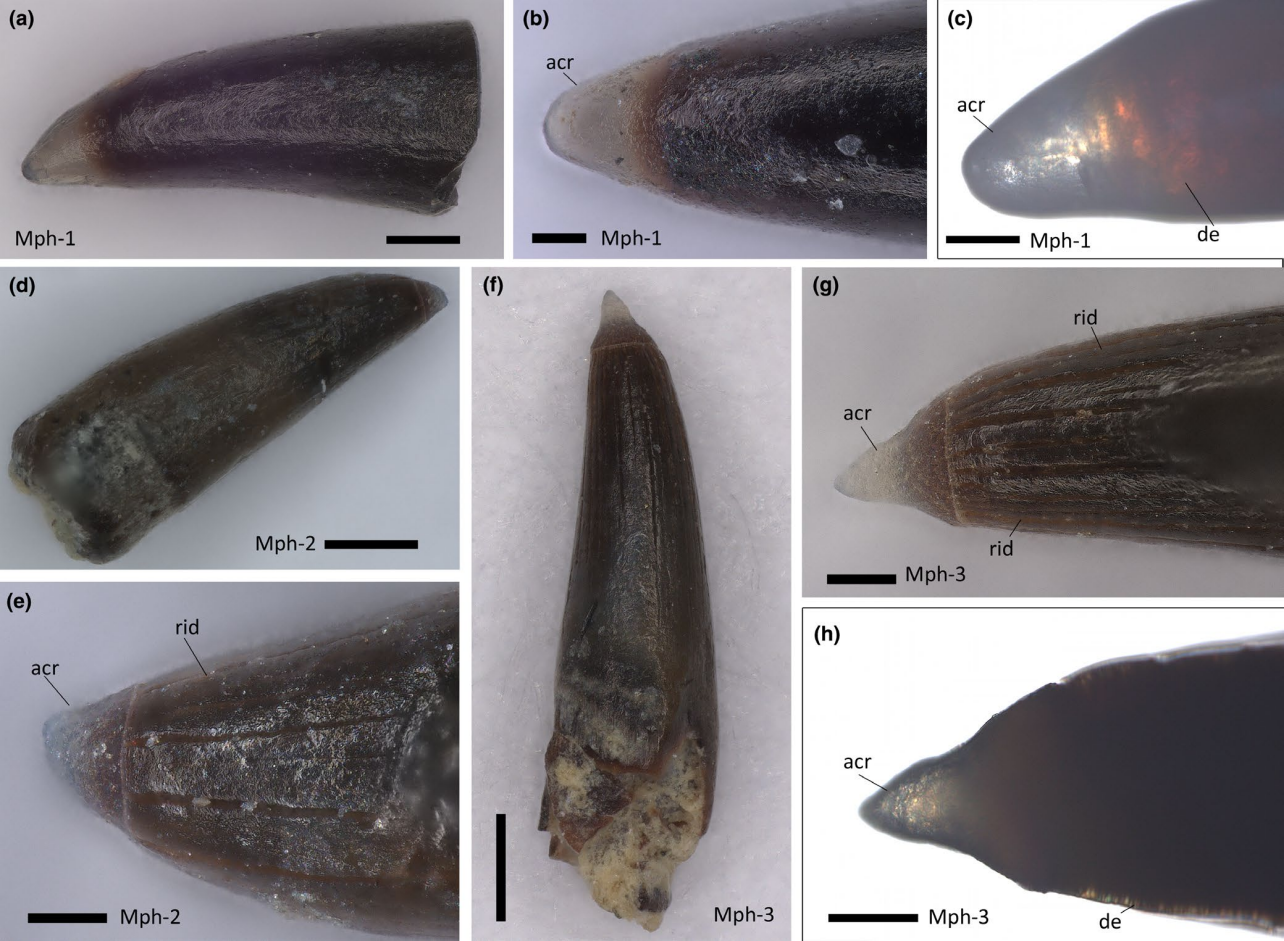
Detention of IPUM 35050. (a) Extracted morphotype 1 tooth. (b) Close‐up of acrodin tip of morphotype 1 tooth. (c) Transmitted light imaging of the acrodin tip of (b). (d) Extracted morphotype 2 tooth. (e) Close‐up of acrodin tip and crown ornamentation of (d). (f) Extracted morphotype 3 tooth. (g) Close‐up of acrodin tip and crown ornamentation of (f). (h) Transmitted light imaging of the acrodin tip of (f). acr, acrodin; de, dentine; mph, morphotype; rid, enamel ridges. Scale bars: (a) 100 μm; (b, c, e, g, h) 50 μm; (d, f) 200 μm.

**FIGURE 6:**
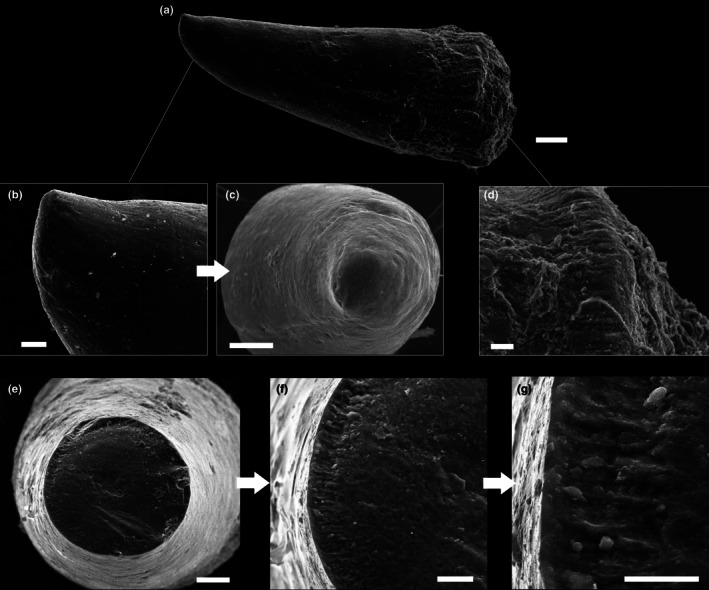
SEM imaging of IPUM 35050 extracted teeth. (a) SEM of entire morphotype 1 tooth. (b) Close‐up of the apical portion of the tooth from (a). (c) Close‐up of the (a) tooth apex in frontal view, with detail of the microwear. (d) Detail of the collar at the base of the tooth from (a). (e) Cracked tip of a tooth. (f) Close‐up of the sectioned tip. (g) Close‐up of the section tip with detail of the radially arranged pillars. Scale bars: (a) 100 μm; (b, d, f) 20 μm; (c, e) 50 μm; (g) 10 μm.

Teeth are generally in the size range between 500 μm and 1 mm. Three dental morphotypes were observed from extracted samples: (1) stout crown with robust shaft and blunt apex, completely smooth in texture (Figure 5a–c); (2) stout and robust crown with faint apicobasal ridges toward the apex (Figure 5d,e); (3) thin crown finely ornamented with apicobasal ridges (Figure 5f–h). All three morphotypes are invariably displaced from their sockets and exhibit a basal ridged collar separating the crown from the attachment bone (Figure 6a,d), suggesting that an unmineralized collagen ring occurred between the tooth base and the bone (Type 2 tooth attachment mode in the sense of Fink, 1981). The preserved teeth are dislodged from the alveoli and randomly oriented. Moreover, all the surveyed teeth show a slight curvature of the crown (of which the direction is difficult to generalize, as lingual and labial topologies lose significance in this dentition) and, most distinctively, an apical capping of translucent acrodin (Figure 5). This capping differs according to relative tooth morphotype, being blunt in Types 1 and 2 whereas it is sharp and basally constricted in Type 3. Under transmitted light, the acrodin layer is easily distinguishable because it lacks any type of ornamentation (Figure 5c,h). Dentine is also appreciable in reddish colors under transmitted light. SEM analysis revealed that the acrodin tips seemingly exhibit signs of micro‐wear (Figure 6b,c). A denticle cracked transversally near the apex displays a peripheral layer organized in radially arranged, micrometric (~10 μm long) pillars (Figure 6f,g).

### 4.4 Internal rostral architecture

The floor of the two premaxillary rami (together with the mesethmoid) constitutes the internal walls of a large (5 cm wide) subtriangular vacuity that progresses longitudinally along the entire upper jaw (Figure 4a–c). Judging from the polished anterior and posterior surfaces and CT data, this large sinus follows the general anterior flattening trend of the upper jaw, showing a slow reduction in height from the posterior (3.9 cm) to the anterior end (2.4 cm). Moreover, this internal vacuity appears to be tripartite by two septa that ventrally enclose two distinct longitudinal “sub‐channels” (Figure 4d–h). We identify these septa as part of the two premaxillae, internally infolding on themselves. When digitally segmented, the central vacuity and the adjacent sub‐canals can be observed running parallel to one another along the entire upper jaw (Figure 4d–h). On the lower jaw, the only anatomical voids are represented by the two Meckelian canals and by the large central cavity made by the enclosing dentaries (Figure 4). No sensory canals or fossae are present on the surface of the preserved dentary rami. From the available CT scan data, no external branchings from the Meckelian canal can be observed.

### 4.5 Osteo‐histological mesostructure

Thanks to the polished ends of IPUM 35050, the bone mesostructure of the specimen can be approximately recognized even at low magnifications. The premaxillary rami are less than 1 cm thick in cross section, with a distinct compact layer on both outer and inner sides, bordering a well‐developed internal cancellous component. Trabecular spaces are relatively large (1 mm in length), giving an alveolate appearance to this tissue (Figure 3c,g,h). The dentigerous layers at the walls and floor of the premaxillae are embedded within the compact bone, appearing as a pitted and darker layer a few millimeters thick (Figure 3f). In the lower jaw, the bone tissue between the outer walls of the dentary and of the Meckelian canal is relatively thick and consistent with the histology of the premaxillae; the bone organization between the inner Meckelian and symphyseal walls appears slightly more alveolated.

### 4.6 Cranial size estimates

In vivo sizes of the specimen are difficult to assess given the fragmentary nature of the partial rostrum. We attempted to provide an approximate estimate of the preorbital snout length and total skull length of the specimen based on scaling of the morphologically similar and possibly taxonomically close *Rhamphoichthys* (see discussion below). Given the uncertainties in positioning IPUM 35050 along the rostrum of the holotype of *Rhamphoichthys taxidiotis* (WMNM P 48342), we compared the results of two measurements: (1) IPUM 35050 maximum length correlated to a 3 cm segment between the maxilla to the beginning of the mandibular symphyseal region of WMNM P 48342 (position based on the presence of a fused mandibular rami in IPUM 35050); (2) IPUM 35050 upper jaw height and the same measure in WMNM P 48342 along the mandibular symphyseal landmark (chosen as relatively consistent along the median‐posterior rostrum of WMNM P 48342). Both scalings have shortcomings, as for Method 1 the positioning of IPUM 35050 along WMNM P 48342 is largely subjective, while for Method 2 we are unable to confirm if the outcropping snout height in WMNM P 48342 truly corresponds to that measured in IPUM 35050. Method 1 resulted in an estimated preorbital snout length of IPUM 35050 of 69 cm, whereas the skull length is reckoned at 92 cm. Method 2 resulted in a preorbital snout length of 58 cm and a total skull length of 77.3 cm. Assuming that *Rhamphoichthys* represents the best anatomical model for IPUM 35050, and given the two different results based both on error‐prone estimates, we find it reasonable to assume the specimen had a preorbital snout length in the 55–70 cm range and a total skull length of 75–90 cm.

### 4.7 Recent analogues comparative analysis: Dentition

**FIGURE 7:**
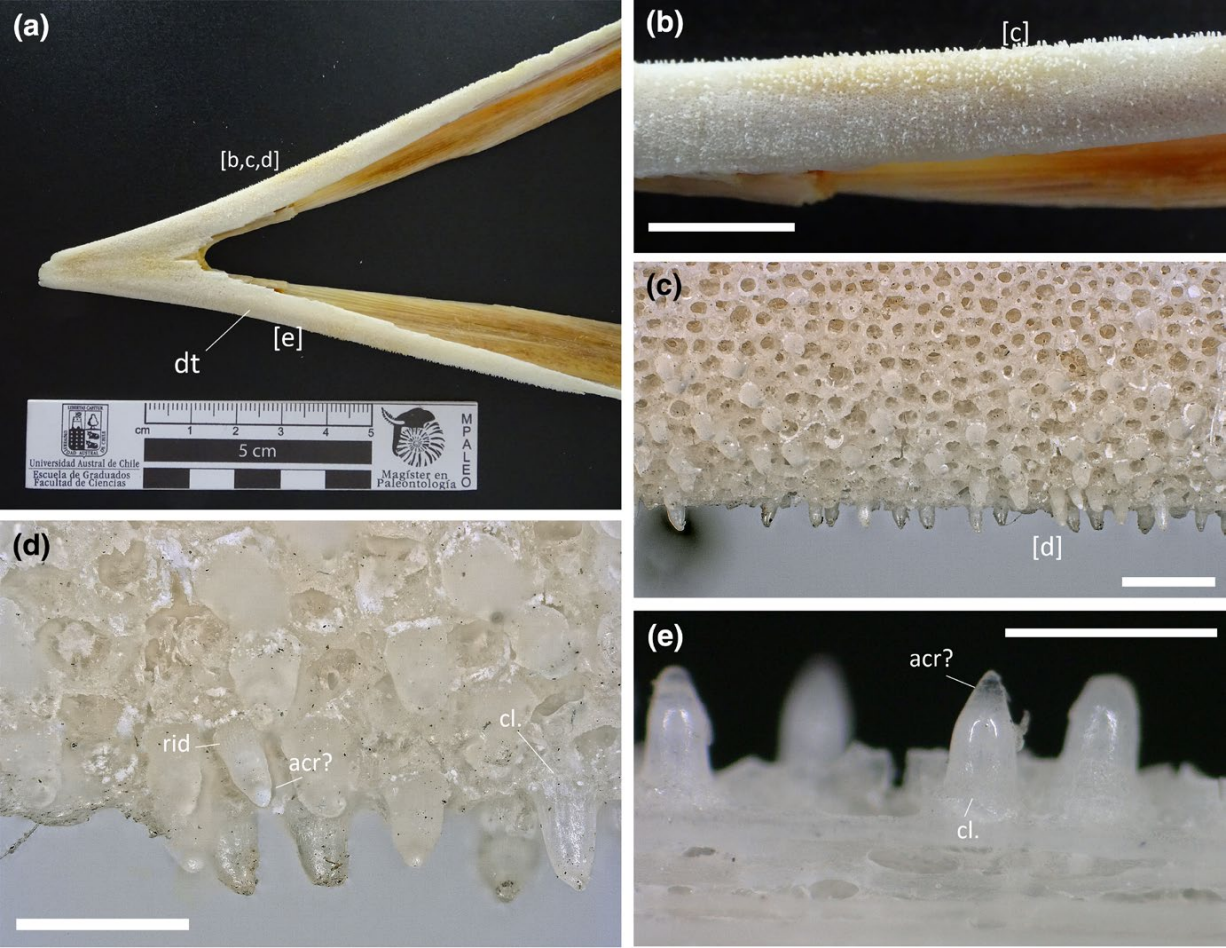
Comparative plate of *Xiphias gladius* dentition. (a) Overview of EMRG‐Act‐A‐82 mandible. (b) Close‐up of the dentary ramus draped in micro‐teeth. (c) Detail of the pitted pediments on the trabecular scaffolding. (d, e) Close‐up of in situ denticles. Letters in brackets correspond to panel positions throughout the specimen. acr, acrodin; cl., collagen ring; rid, ridges. Scale bars: (b) 1 cm; (c) 1 mm; (d, e) 5 mm.

The tiny teeth of IPUM 35050 were directly compared to those of a Recent swordfish *Xiphias gladius* to better frame morphological similarities. As micrographs of xiphiid dentitions have been rarely figured in recent literature (Häge et al., 2022), we report herein our results for comparison and characterization. Teeth were analyzed from the post‐symphyseal region of a 22‐cm long mandible from a Mediterranean *X. gladius* (EMRG‐Act‐A‐82; Figure 7a). Numerous teeth are associated with small sockets that drape the outer margin of the alveolar surface of the dentary (Figure 7b). There is a ring of collagen between the base of the teeth and the attachment bone (Type 2 mode of tooth attachment in the sense of Fink, 1981) (Figure 7c–e). These small sockets were described as “pediments” by Carter (1919), subepithelial structures at the dermal junction attached to what he describes as a “translucent zone” which itself overlays a trabecular network. It is interesting to note that the dentigerous areas in the premaxilla and dentary of IPUM 35050 also overlay a similar trabecular‐like organization in the form of the reticular ornamentation of the bone surface (Figure 7c–e). The sockets of IPUM 35050 (analogues to *Xiphias* pediments) appear more regularly shaped and sized than they are in the extant swordfish. The teeth of *Xiphias gladius* (EMRG‐Act‐A‐82) are conical in shape and share the same orientation of their apicobasal curvature. They are translucent as the socket and the underlying trabecular scaffolding, but some of the smaller, possibly newly erupting teeth also exhibit a white, opaque acrodin capping on the tip (Figure 7d,e). Finally, while larger teeth in EMRG‐Act‐A‐82 appear completely smooth and with a blunt apex, some smaller elements do present fine longitudinal ridges running from the base to the midline of the tooth (Figure 7d). This variation in tooth ornamentation is also consistent with the presence of different morphotypes in IPUM 35050.

### 4.8 Recent analogues comparative analysis: Rostral sinuses

**FIGURE 8:**
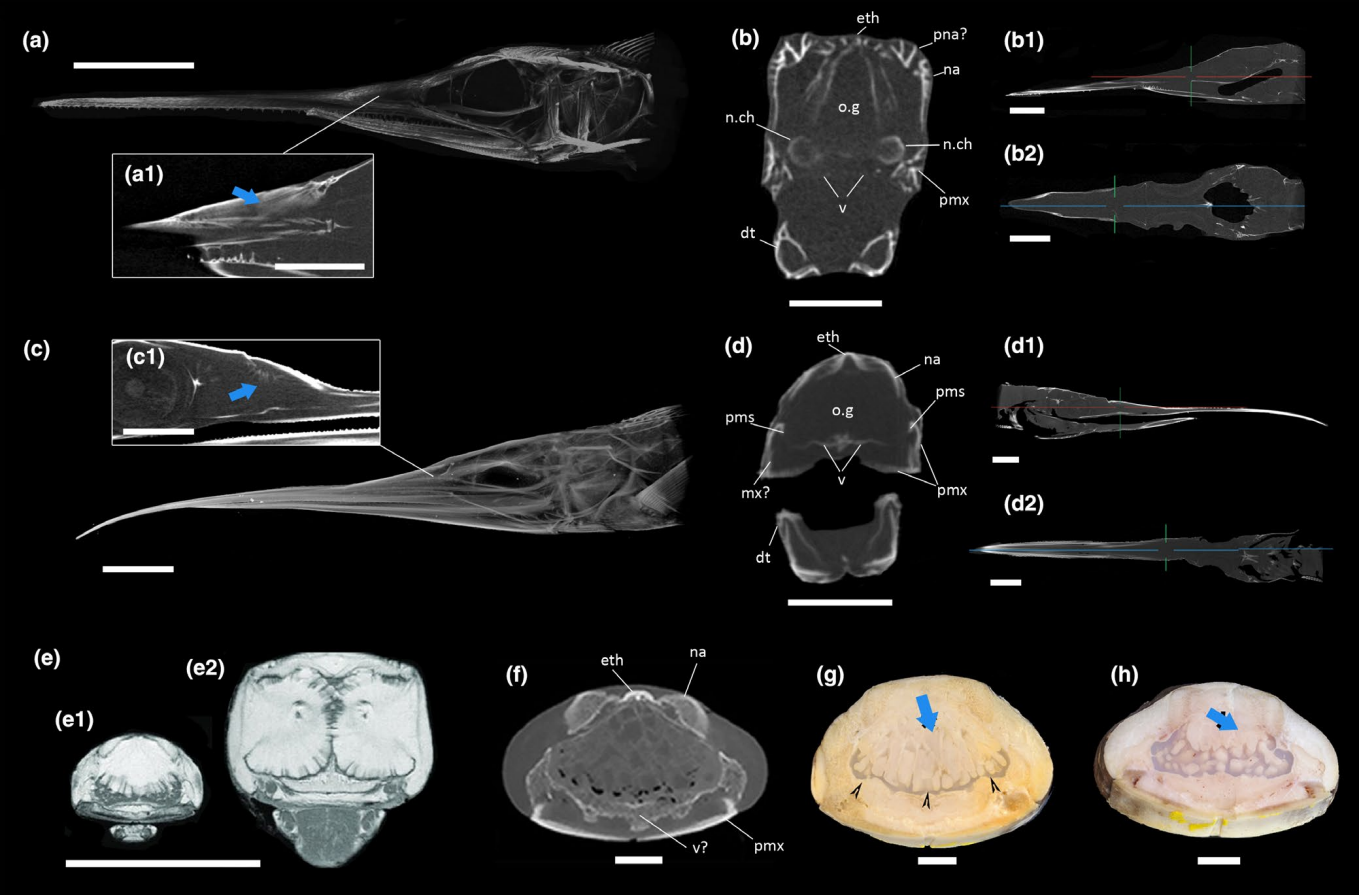
Comparative plate on rostral sinuses of extant xiphioids. (a) Volumetric rendition of postlarval *I*. *platypterus* CT scan, with detail (a_1_) on oil gland texture. (b) CT slices from the same spot of specimen from (a) in axial, lateral (b_1_) and dorsal (b_2_) view. (c) Volumetric rendition of postlarval *X*. *gladius* CT scan, with detail (c_1_) on oil gland texture. (d) CT slices from the same spot of specimen from (c) in axial, lateral (d_1_), and dorsal (d_2_) view. (e) MRI imaging of an adult *X. gladius* in anterior (e_1_) and posterior (e_2_) axial slices, modified from Videler et al. (2016), reproduced with permission. (f) CT scan of an adult *X*. *gladius* mid rostrum with detail to the osteology surrounding the oil gland sinus. (g) Anatomical section of the posterior‐mid portion of the rostrum in (f). (h) Anatomical section of the mid‐anterior portion of the rostrum in (f). (f) to (h) are modified from Pazzaglia et al. (2022), reproduced with permission. dt, dentary; eth, ethmoid; mx, maxilla; na, nasal; n.ca, nutrient canal origin; o.g, oil gland sinus; pm.s, premaxillary septum; pna, prenasal; pmx, premaxilla; v, vomer. Scale bars: (a, c_1_, d, e) 5 mm; (a_1_), 1 mm; (b_1_), 3 mm; (b_2_), 2 mm; (c, d_1_, d_2_, g, h, f) 1 cm; (e) 5 cm. Comparative CT data (UF‐Fish 171,758 Head ark:/87602/m4/501620; UF‐Fish 187,897 Head ark:/87602/m4/M167333) were provided by the Florida Museum of Natural History (FLMNH), Division of Ichthyology, data deposited by Zach Randall. The files were downloaded from www.MorphoSource.org, Duke University).

The internal architecture of the upper rostrum of IPUM 35050 was directly compared with those of modern billfishes. Tall, sub‐triangular vacuities associated with oil glands and fat stroma (see discussion below) can in fact be observed in the posterior upper jaw of both members of Xiphiidae and Istiophoridae (e.g., Videler et al., 2016). In addition to adult individuals sourced from available literature, we surveyed these rostral sinuses in late postlarvae of *Istiophorus platypterus* (sailfish; Figure 8a,b) and *Xiphias gladius* (swordfish; Figure 8c,d). The comparison between IPUM 35050 and early staged billfishes is fitting, as both share a similar rostral architecture with relatively long lower jaws. Both the surveyed postlarval *I. platypterus* and *X. gladius* specimens exhibit well‐developed rostral sinuses. At the base of the rostral cone, these chambers are tall, sub‐rectangular in *I. platypterus* (Figure 8b) and more triangular in *X. gladius* (Figure 8d). These sinuses develop prenarially and persist anteriorly at about half of the rostrum. CT scan slices allow us to identify fibrous infilling of these vacuities, consistent with previously described MRI and CT imaging of oil glands (Pazzaglia et al., 2022; Videler et al., 2016). The oil gland sinuses are floored by palatal bones, with a vomeral plate ventral to each lobe; this aspect contrasts with the condition described in IPUM 35050, where the analogous position lacks any discernible palatal component. We note, nonetheless, that the anatomical position of the fragmentary specimen IPUM 35050 might not correspond exactly to the analyzed sections from extant xiphioids, thus resulting in some expected minor osteological differences. Laterally, premaxillae enclose the sinus, whereas dorsally either the nasals (*X. gladius*) or the prenasals (*I. platypterus*), together with the ethmoidal bone, encompass the roof of the glandular chamber. The lateral lobes of the oil gland of the postlarval sailfish are arranged around two longitudinal canals that anteriorly pass to the rostral “nutrient canals” (e.g., Fierstine, 2006; see discussion below), a characteristic already described in adult specimens of *X. gladius*. Interestingly, the postlarval swordfish seems to show internal infoldings of the premaxillae in axial sections (Figure 8d), a feature not dissimilar to the premaxillary septa described in IPUM 35050 (though much smaller). Overall, the rostral sinuses architecture in postlarval *Xiphias* is partially consistent with that of the adults, indicating that the oil glands (or at least the sinuses they reside in) develop early during the ontogeny (Figure 8e,f). The same apparently occurs in *Istiophorus*, though less information on the gross morphology/histology of oil glands is available for adults. Moreover, thanks to both internal imaging and histological sections, we can recognize a distinct osteological correlate for the presence of oil glands and associated fat stroma, as these glandular structures are consistently enclosed in tall and angulated chambers in posterior sections of the rostral cone (Figure 8g,h).

## 5 DISCUSSION

### 5.1 Taxonomic assignment

Given the fragmentary nature of the specimen with little osteological material preserved, a genus‐ or species‐level taxonomic assignment for IPUM 35050 cannot be assessed. Due to its Cretaceous age and the presence of a rostral mesethmoid rather than paired nasals/prenasals, the alignment to the extant longirostrine Istiophoridae and Xiphiidae can be ruled out. Similarly, the osteology of the rostrum and the reductive dentition of the specimen are not consistent with those of *Protosphyraena* (Kanarkina et al., 2025; Woodward, 1895) as well as those of the Cretaceous members of the family Aspidorhynchidae (e.g., Brito, 1997). However, IPUM 35050 shares the presence of small teeth covering the jaws and the presence of tooth‐bearing elements pierced by small and numerous pits with the tselfatiiform family Plethodidae (e.g., Taverne & Gayet, 2005). Pitted dentigerous elements have been reported for the enigmatic plethodid *Martinichthys* (McClung, 1926) as well as in other better‐known genera, such as *Tselfatia*, *Dixonanogmius* (Taverne & Gayet, 2005), and *Pentanogmius* (Taverne, 2000b). Moreover, IPUM 35050 also exhibits an elongated snout forming a strong rostrum in which the two elongated premaxillae are joined ventrally, creating a false palate; this configuration has been considered autapomorphic of *Martinichthys* by Taverne and Gayet (2005). Although fragmentary, IPUM 35050 is here inferred to possess a rostrum much longer than that of *Martinichthys*, a feature proportionally similar to the condition reported in the highly specialized longirostrine plethodid *Rhamphoichthys* from the Cenomanian of Germany and Lebanon (El Hossny et al., 2023). Moreover, *Rhamphoichthys* is described as having a hollow upper‐jaw rostrum, but this condition is observed only towards the tip (El Hossny et al., 2023). IPUM 35050 (although anteriorly incomplete) seemingly lacks the characteristic dorsoventral flattening of the rostrum seen in *Rhamphoichthys*. We therefore assign IPUM 35050 to an indeterminate Plethodidae based on rostral and dental similarities with members of this family. Given its large size and deep, cylindrical jaws (features never previously reported in other plethodids), IPUM 35050 likely represents a new taxon. However, we are unable to produce a reliable diagnosis for the creation of a new genus and/or species due to the fragmentary nature of the specimen. IPUM 35050 appears to be in some ways similar to *Martinichthys* and *Rhamphoichthys*. El Hossny et al. (2023) tentatively referred the Italian specimens of “*Protosphyraena” stebbingi* to the genus *Rhamphoichthys*. Similarly, “*P.” minor* from the English Chalk is also considered to share many similarities with *Rhamphoichthys*. These dorso‐ventrally flattened isolated rostra were previously attributed to an indeterminate tselfatiiform by Amalfitano et al. (2019). However, while refraining here from discussing this attribution in detail, we conclude that IPUM 35050 and these elongated tselfatiiform rostra do not belong to the same taxon due to both odontoskeletal and stratigraphical differences. IPUM 35050 is in fact larger and differently shaped from “*P.” stebbingi* and “*P.” minor* (premaxiilary rami cylindrical in IPUM 35050 vs. dorsoventrally flattened in “*P.” stebbingi/*“*P.” minor*), exhibits a different ornamentation (reticular in IPUM 35050 vs. longitudinally striated/smooth [and, in some rostral portions, serially pitted] in “*P.” stebbingi/*“*P.” minor*) and presents evident dental alveoli at the floor of the premaxilla which are absent in “*P.” stebbingi and “P.” minor*, although these might have been present in the non‐preserved, more posterior portion of the rostrum. Lastly, IPUM 35050 is at least 20 My younger than these enigmatic longirostrine taxa, even though we acknowledge that stratigraphic position might not be a good taxonomic criterium.

### 5.2 Rostral anatomy in plethodids

As discussed above, some members of the family Plethodidae exhibit a prominent snout, but only a few taxa are characterized by an elongate rostrum. The elongation, morphology and degree of fusion between the elements of the snout varies, but the overall skeletal architecture is consistent among all the taxa. The premaxillae form a rostrum that is integrated with the neurocranium. The premaxillae are completely fused along the midline in the anterior part of the rostrum. In the posterior part, a longitudinal suture divides the posterior portion of the premaxilla from the mesethmoid, which forms the base of the rostrum. Dorsally, a small portion of the mesethmoid is exposed and separates the premaxillae from the anterior tips of the frontals, thus connecting the rostrum to the skull roof. Among all plethodids, *Martinichthys*, *Thryptodus*, and *Rhamphoichthys* are the only genera with elongated fused premaxillae forming a rostrum, with *Rhamphoichthys* showing the more extreme elongation (El Hossny et al., 2023). Taverne (2000a) recognized only two valid species of *Martinichthys*: the type species, *M*. *brevis* and *M*. *ziphioides*. These species differ mostly in proportions of the snout, with that of *M*. *ziphioides* being longer and thinner than that of *M*. *brevis* (El Hossny et al., 2023; Taverne, 2000b). The morphology of *Rhamphoichthys* and *Martinichthys* rostra differs: the more modest rostrum of the latter is much thicker and more rounded compared to that of *R. taxidiotis*, which has a narrower rostrum ending into a pointed to slightly rounded tip (El Hossny et al., 2023). The rostrum of *R. taxidiotis* is also dorsoventrally flattened, with a midline groove on the ventral sides and raised edges. This is markedly different from the condition of IPUM 35050, which, at least in the mid‐posterior portion of the rostrum, does not exhibit substantial flattening nor a grooved surface of the false palate. The surface ornamentation of *Rhamphoichthys* exhibits parallel longitudinal and anastomosing ridges, with different orientation along the lateral walls (such as those of *Protosphyraena stebbingi* and “*P*.” *minor*), which contrasts with the densely pitted cases in *Martinichthys* and other plethodids (El Hossny et al., 2023; Shimada, 2017; Taverne, 2000b; Taverne, 2003). The reticular ornamentation of IPUM 35050 differs from both of these conditions. The sutures between the elements of the rostrum of *Martinichthys* are less evident, indicating a more pronounced fusion than those of *Rhamphoichthys* and IPUM 35050. *Thryptodus* is another taxon characterized by a relatively prominent rostrum although blunter in comparison to those of *Martinichthys* and *Rhamphoichthys* (Shimada, 2017; Taverne, 2003). It also exhibits morphological variation through ontogeny, starting from a gently pointed tip in young individuals becoming blunter and more squared in older specimens (Shimada, 2017). Fusion of the elements of the rostrum seemingly increases during ontogeny, with a progressive obliteration of the sutures that allow to identify the different bones. The function of the robust rostrum in *Thryptodus* remains uncertain (Shimada, 2017). The fossae identified at the level of the suture between the premaxillae and the mesethmoid in IPUM 35050 have no analogue in other plethodids.

### 5.3 Dentition in plethodids

**FIGURE 9:**
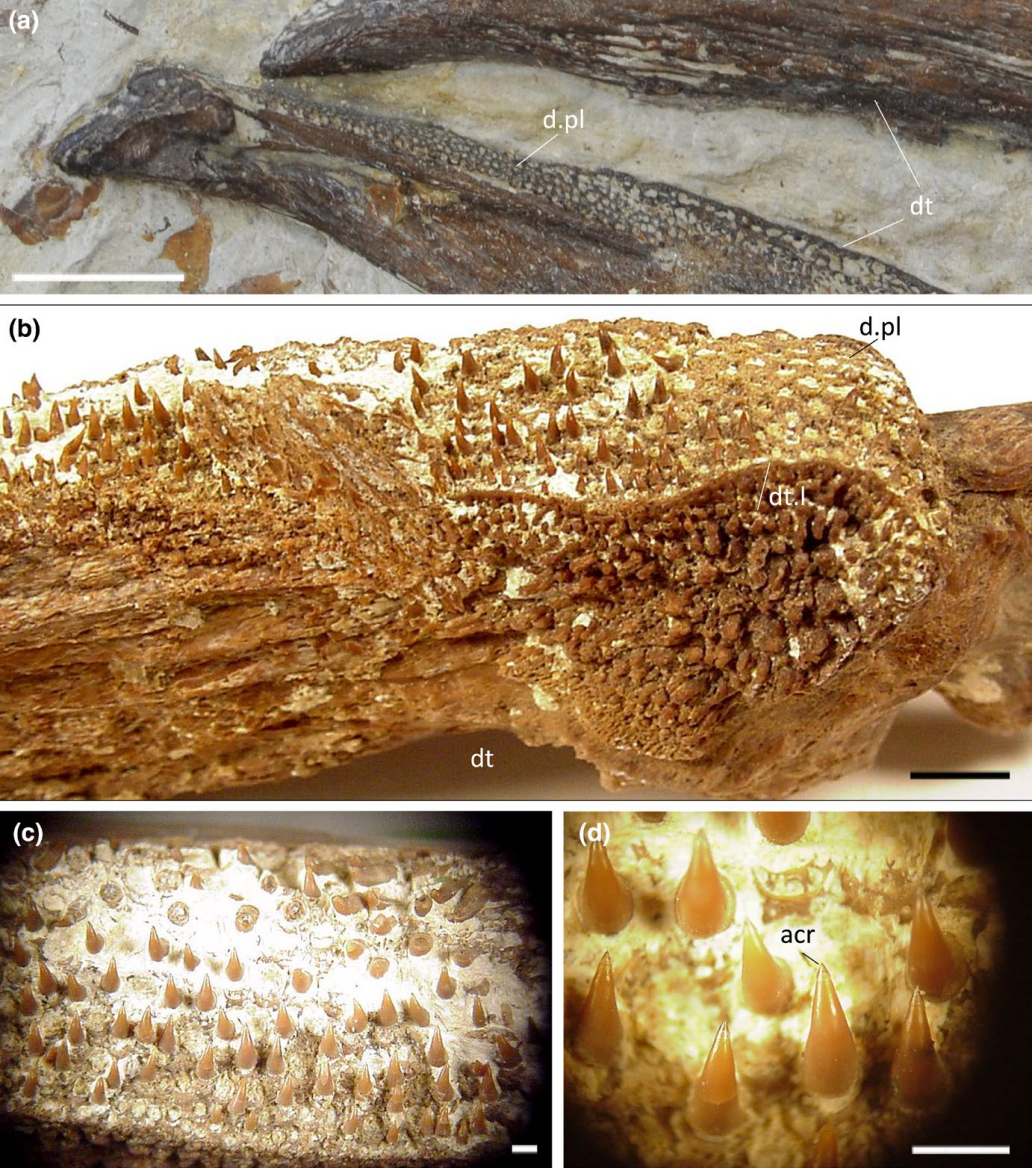
Comparative plate on plethodid dentition: (a) Dentary tooth plate from the holotype of *Rhamphoichthys* (WMNM P 48342; picture courtesy of Tamara El Hossny, reproduced with permission of Matt Friedman). (b) Dentary of *Pentanogmius evolutus* with details of the villiform teeth. (c, d) Close‐ups of maxillary teeth of *P*. *evolutus*. Panel (b) to (d) courtesy of Mike Everhart. acr., acrodin; d.pl., dental plate; dt, dentary; dt.l, dentigerous layer. Scale bars: (a) 1 cm; (b) 3 mm; (c, d) 1 mm.

The structure of the teeth of IPUM 35050 is generally similar to what has been cursorily reported for other plethodids. Members of the Plethodidae have been consistently described with rounded and tightly packed sockets (Figure 9a) arranged in distinctive plates or bands (e.g., Fielitz & Shimada, 1999; Stewart, 1900; Taverne & Gayet, 2005), whereas teeth are typically referred to as “villiform” or small and “comb‐like” in structure (El Hossny et al., 2023; Everhart, 2005). As we provide for the first time microscopic photographic documentation of plethodid teeth, it is difficult to provide a detailed comparative analysis involving the other plethodid taxa. Precise dental measurements are unavailable from the literature; however, we note that the teeth of IPUM 35050 appear to be more slender than those figured for the other taxa (e.g., *Pentanogmius evolutus*; Taverne, 2004). Herein, we figure the only other solely available microphotograph of a plethodid dental plate with associated teeth from a *Pentanogmius* sp. specimen (courtesy of M. Everhart), in which teeth are characterized by morphological attributes (orientation and curvature; Figure 9b–d) fully consistent with those observed in modern xiphioids. Additionally, xiphioids also exhibit micro‐teeth organized in plates and/or bands in some taxa (Schultz, 1987). Moreover, small translucent cappings can be distinguished on the dental tips (Figure 9c,d), in some cases seemingly constricted towards the apex as in Morphotype 3 of IPUM 35050. A similar capping has also been identified in isolated plethodid teeth (VP‐15224, T. El Hossny, personal communication; S2).

### 5.4 IPUM 35050 rostral vacuity: A glandular sinus?

A striking feature of the upper jaw architecture of IPUM 35050 is its large triangular vacuity subdivided by premaxillary septa into a larger central and two adjacent smaller canals (Figure 4). A similar space is present at the beginning of the rostrum of modern xiphioids, including *Xiphias gladius*, *Istiophorus platypterus*, *Kajikia audax*, and *Makaira nigricans* (e.g., Dhellemmes et al., 2020; Pazzaglia et al., 2022). MRI, CT scan analyses, and histological sections on extant xiphioid taxa have confirmed that these large antorbital spaces in the upper rostrum are occupied by oil glands, which are globose glandular structures inserted in a cartilaginous scaffolding (Pazzaglia et al., 2022; Videler et al., 2016). *Xiphias gladius* oil glands are composed of two large, symmetric lobes immediately in front of the eyes that develop radially around the two paired neurovascular channels of the rostral cone (Videler et al., 2016; Figure 8e). These canals are filled with an adipose stroma and crossed by arteries and myelinic nerves (Pazzaglia et al., 2022), and progress longitudinally along the entire rostrum length. These canals are well known in extant and fossil billfish rostra, often referred to as “nutrient canals” (e.g., De Gracia et al., 2022; Fierstine & Voigt, 1996). Anterior to the paired lobes, the oil gland of *Xiphias gladius* is described by Pazzaglia et al. (2022) to reduce in size, occupying a triangular cavity floored by a cartilage‐like matrix (Figure 8g); with the distal flattening of the rostrum, the glandular tissue and cartilaginous matrix reduce until their disappearance from the progressively more calcified rostral core (Pazzaglia et al., 2022). These oil glands are connected to the head surface via a minute capillary network extending from the base of the rostrum to the opercula, opening to the epidermis through distinctive skin pores (Dhellemmes et al., 2020; Videler et al., 2016). The terms “*glandula oleofora*” and “*rete lubricans*” were proposed by Videler et al. (2016) to identify these structures. The oily secretion is mainly composed of docosahexaenoic, oleic, and palmitic acids, fatty compounds believed to acquit both a hydrodynamic purpose in reducing the drag of the head during fast swimming and an anti‐inflammatory function during traumatic activities of the bill/spear (Dhellemmes et al., 2020). The anti‐inflammatory hypothesis is consistent with results from biomechanical and osteohistological studies of the billfish rostrum, where torsional stress resistance, coupled with active bone remodeling, suggests a dynamic and injury‐accustomed use of the organ (Atkins et al., 2014; Habegger et al., 2020). Additional (and non‐mutually excluding) hypothesis for the function of oil glands and secreted fatty acids in extant billfishes were proposed by Dhellemmes et al. (2020), including rostral shock absorption, eyes insulation for sight improvement, and aid in maintaining positive buoyancy of the fish body.

The large void in the upper jaw of IPUM 35050 could have housed a structure similar to a *glandula oleofora* based on comparative anatomy with modern analogues and the relative position of the fragment along the rostrum length. The central void has a sub‐triangular shape, reminiscent only of the more anterior portion of the oil gland rostral sinus in modern xiphioids. Other longirostrine fishes that exhibit a hollow rostrum lacking oil glands have, in fact, different architectures of the central vacuity, like the axial duct in pristid sawfishes (small, laterally constricted in cross section; Wueringer et al., 2009) or the hollow snout of Acipenseriformes (rounded chamber in cross section with thin bony walls sporting lateral flanges; e.g., *Polyodon spathula*; Humphries, 2009). Moreover, we highlight how the alveolated histology of the rostral bones in IPUM 35050 appears strikingly similar to the cancellous‐like thin laminae and septa reported by Pazzaglia et al. (2022) in the outer portion of *Xiphias gladius* bones associated with fat stroma. Given the incompleteness of the specimen, we are unable to verify whether the two adjacent linear sub‐canals separated from the main central vacuity by premaxillary septa could have progressed in true neurovascular canals (=nutrient canals), but this interpretation is likely. Given the similar macro‐histology of these subsidiary canal walls to those of the main central cavity, these structures would have likely been filled by a fat stroma. Unfortunately, we are unable to trace the vascular pathway of an analogous *rete lubricans* in the specimen, as the dense mineral permeation hampers the detection of fine branching channels from the central voids to the outside. We note, however, that the longitudinal thin canal adjacent to the premaxillary fossa appears to be very close to these putative oil gland sinuses (Figure 4d,f), being possibly associated with those by unseen vascular connections. Moreover, the grooves reported at the floor of the mesethmoid could also represent connective pathways for such secretion. Waiting for the recovery of more complete specimens, we speculate that these mesethmoid grooves and fossa‐adjacent canals might be involved in oil distribution on the rostrum surface, among other functions (e.g., neurovascular pathway).

### 5.5 Craniodental convergences

IPUM 35050 exhibits a reduced lower jaw relative to the robust upper jaw, a cranio‐mandibular morphology consistent with many longirostrine actinopterygian taxa, especially post‐Neogene xiphioids (Fierstine, 2006; Fierstine & Voigt, 1996; Habegger et al., 2020). Moreover, the partial rostrum of IPUM 35050 shows a gentle antero‐posterior flattening of both cranium and mandibles. However, while in most extant xiphioids this flattening trend is more pronounced (Fierstine, 2006), IPUM 35050 exhibits a more rounded cross section of the rostrum (Figure 2c,d). Some billfishes show a relatively deep rostral morphology, including the Eocene blochiid *Loancorhynchus* (Otero, 2019), the Eocene–Oligocene paleorhynchid *Aglyptorhynchus* (Fierstine, 2001, 2005, 2006), the Eocene–Oligocene *Xiphiorhynchus* (Monsch, 2004), the Miocene istiophorid *Morgula donosochagrense* (De Gracia et al., 2022), as well as the istiophorid *Makaira* spp. (De Gracia et al., 2022; Fierstine, 2006). A deeper and more robust bill shape is generally regarded as more suited for a multiplanar swiping motion rather than a slashing one, a functional morphology of the rostrum supported also by finite elements‐based biomechanical studies (Domenici et al., 2014; Habegger et al., 2020; Hansen et al., 2020). Similar morpho‐functional insights have been also proposed for fossil specimens (De Gracia et al., 2022). This feature might suggest a similar hunting strategy for IPUM 35050's taxon, though its more conical jaws differs also from most deep‐snouted istiophorids. The ratio between proximal rostral height and width (D1/W1 sensu Fierstine & Voigt, 1996, originally designed only for xiphioids) is indicative of deep‐snouted taxa, reaching values closer to 1. A ratio of 1 implies a perfectly circular cross‐section of the upper jaw rostrum, a condition seemingly only achieved by *Protosphyraena* spp. (Kanarkina et al., 2025). When analyzing snout ratios in different longirostrine teleosteomorphs (see Table 1), IPUM 35050 scores a value of 0.85. This ratio is slightly higher than the one of extant *Makaira*, *Istiompax*, *Istiophorus Kajikia*, and *Prototetrapturus*, as well as most Neogene istiophorids (e.g., *Makaira fierstini*, *Ma*. *belgica*, *Morgula*, and *Spathochoira*; De Gracia et al., 2022). Interestingly, the rostral ratio of IPUM 35050 is also higher than the one of the presumably related Cretaceous plethodid *Rhamphoichthys* (Table 1). A more similar D1/W1 ratio is shared by Paleogene xiphioids, such as *Xiphiorhynchus rotundus* (McCuen et al., 2020) and *Aglyptorhynchus* spp., together with the Cretaceous plethodid *Martinichthys* (McClung, 1926) and the pachycormid *Protosphyraena* spp. (Kanarkina et al., 2025; Table 1).

**TABLE 1:** Rostral dimensions from longirostrine actinopterygians discussed in this study (Pachycormidae, Plethodidae, and Xiphioidea).

	Family	Epoch	Rostrum height/width	Rostrum length (mm)	References
*†P. tenuirostris* (*n* = 2)	Pachycormidae	Upper Cretaceous	1	244	Woodward (1895); Kanarkina et al. (2025)
*†P. ferox* (*n* = 2)	Pachycormidae	Upper Cretaceous	1	290	Woodward (1895); Kanarkina et al. (2025)
*†R. taxidiotis* (*n* = 2)	Plethodidae	Upper Cretaceous	0.75	180	El Hossny et al. (2023)
*†’P′. stebbingi* (*n* = 1)	?Plethodidae	Upper Cretaceous	0.30	461	El Hossny et al. (2023)
*†M. brevis* (*n* = 1)	Plethodidae	Upper Cretaceous	0.83	43	McClung (1926)
*†M. ziphioides* (*n* = 3)	Plethodidae	Upper Cretaceous	1.17^a^	90	McClung (1926)
*†*IPUM 35050 (*n* = 1)	Plethodidae	Upper Cretaceous	0.85	550–700	This study
*†L. catrillancai* (*n* = 1)	Blochiidae	Eocene	0.55	NA	Otero (2019)
*†Xi. eocenicus* (*n* = 1)	Xiphiidae	Eocene	0.60	NA	Monsch (2004)
*†Xi. aegyptiacus* (*n* = ?)	Xiphiidae	Eocene	0.45	NA	McCuen et al. (2020)
*†Xi. rotundus* (*n* = ?)	Xiphiidae	Eocene?	0.84	NA	McCuen et al. (2020)
*†A. maxillaries* (*n* = 1)	Palaeorhynchidae	Oligocene	0.81	352	Fierstine (2001)
*†A. columbianus* (*n* = 1)	Palaeorhynchidae	Oligocene	0.83	NA	Fierstine (2005)
*†Mo. donosochagrense* (*n* = 1)	Istiophoridae	Miocene	0.73	624	De Gracia et al. (2022)
*†Ma. fierstini* (*n* = 1)	Istiophoridae	Miocene	0.75	794	De Gracia et al. (2022)
*†Ma. colonense* (*n* = 1)	Istiophoridae	Miocene	0.70	672	De Gracia et al. (2022)
*†Ma. belgica* (*n* = 1)	Istiophoridae	Miocene	0.69	430	De Gracia et al. (2022)
*†S. calvertense* (*n* = 1)	Istiophoridae	Miocene	0.71	551.2	De Gracia et al. (2022)
*†Pr.courcelli* (*n* = 1)	Istiophoridae	Miocene	0.66	580	De Gracia et al. (2022)
*I. platypterus* (*n* = 31)	Istiophoridae	Recent	0.68	502.2	Fierstine and Voigt (1996); De Gracia et al. (2022)
*Ma. nigricans* (*n* = 40)	Istiophoridae	Recent	0.70	588.3	Fierstine and Voigt (1996); De Gracia et al. (2022)
*Is. indica* (*n* = 7)	Istiophoridae	Recent	0.73	611.7	Fierstine and Voigt (1996); De Gracia et al. (2022)
*K. audax* (*n* = 12)	Istiophoridae	Recent	0.68	540.4	Fierstine and Voigt (1996); De Gracia et al. (2022)
*K. albida* (*n* = 15)	Istiophoridae	Recent	0.58	464.4	De Gracia et al. (2022)
*T. angustirostris* (*n* = 3)	Istiophoridae	Recent	0.64	NA	Fierstine and Voigt (1996); De Gracia et al. (2022)
*X. gladius* (*n* = 40)	Xiphiidae	Recent	0.34	NA	Fierstine and Voigt (1996)

*Note*: Blue boxes refer to Cretaceous non‐xiphioid taxa that independently acquired a longirostrine condition, red boxes refer to true xiphioids. Genera abbreviations: A., *Aglyptorhynchus*; Is., *Istiompax*; I., *Istiophorus*; K. *Kajikia*; L., *Loancorhynchus*; Ma. *Makaira*; M., *Martinichthys*; Mo., *Morgula*; P, *Protosphyraena*; Pr., *Prototetrapturus*; R., *Rhamphoichthys*; S., *Spathochoira*; T., *Tetrapturus*; X., *Xiphias*; Xi., *Xiphiorhynchus*. *†*, extinct taxon; *n*, number of specimen (for *n* > 1 a mean was applied); NA, non avaliable. Rostral height‐width ratio follows Fierstine Voigt (1996) and De Gracia et al. (2022), but the same measurements at the proximal end of the rostrum (D1; W1 sensu Fierstine & Voigt, 1996) are approximated from non xiphioid specimens.

^a^ High ratio possibly due to compression of the specimen (McClung, 1926).

Though fragmentary, we can hypothesize that the mandibles of IPUM 35050, when complete, should have been relatively long: the dorsal fusion of the two dentary rami (which generally anticipates the closure and distal end of the mandibles in billfishes) appears in fact relatively foregone in IPUM 35050, likely indicating an anterior persistence of the lower jaws along the mid rostrum. This condition differs from both the plethodid *Rhamphoichthys* and most of the analogues xiphioids, but with Palaeorhynchidae and *Xiphiorhynchus* spp. being notable exceptions with lower jaws as long as the upper ones (Fierstine, 2006).

However, the most peculiar character of IPUM 35050 in terms of cranial convergence with other longirostrine marine vertebrates is the presence of a longitudinal fossa in the premaxilla. While extant xiphioids are characterized by shallow grooves at the base of the rostral cone in correspondence to the sutures (e.g., maxilla‐nasals), they drastically differ from the long and deep canals of IPUM 35050. This feature bears remarkable similarities to the *fossa premaxillaris* of ichthyosaur rostra. Parvipelvian ichthyosaurs (Diapsida *incertae sedis*) share the presence of a deep longitudinal groove crossed by premaxillary foramina (Maisch, 1998); both fossae and foramina are functionally associated with the rostral neurovascular network (Serafini et al., 2022). Being the fossae of IPUM 35050 also associated with neighboring canals, we suggest that the structure perhaps served in the branching of vascular, nervous, and/or glandular network to the elongated rostrum surface. This shared rostral feature is extremely interesting in highlighting patterns of cranial convergences between amniotes and non‐amniotes in the marine realm. We note nonetheless that the fossa in the surveyed specimen differs from a true ichthyosaurian *fossa permaxillaris*, being localized at the suture between two bones and not as an infolding of the sole premaxillary bone. Moreover, while ichthyosaurs show a similar structure in the lower jaw (*fossa dentalis*), this is seemingly absent in IPUM 35050.

The teeth of IPUM 35050 are remarkably similar to the mandibular dentition of *X. gladius* surveyed in this study. Teeth are approximately consistent in size, and they share a similar conical shape with gentle apico‐basal curvature (Figures 5, 6, 7). The Type 2 mode of tooth attachment of IPUM 35050 is also consistent with the attachment site of the tooth “pediment” described for *X. gladius* and *I*. *platypterus* (Carter, 1919). The comparative analysis of *X. gladius* revealed that smaller and younger (i.e., newly erupting) micro‐teeth are characterized by faint apicobasal ridges, whereas larger and older teeth appear smooth and blunter; this differential ornamentation during dental growth might explain the presence of three distinctive tooth morphotypes in IPUM 35050. We hypothesize that the different tooth morphotypes described in this plethodid specimen correspond to different growth stages showing a pattern similar to that of modern xiphioids rather than representing a functional heterodonty, as they come from the same area of the rostrum, or serve a sensory function since they are not associated with istiophorid‐like *lacunae rostralis* (Häge et al., 2022). Finally, we note that the tiny teeth of *X. gladius* and IPUM 35050 share a similar acrodin capping.

Lastly, as discussed above, comparative analysis of the internal rostral anatomy of IPUM 35050, swordfishes, and billfishes demonstrates a remarkable convergence in the internal architecture between the three taxa. Based on the comparative anatomy of modern xiphioids, the tall central sinus in the upper jaw of IPUM 35050 likely correlates with the hosting of a large and globose oil gland.

### 5.6 Body size and ecology of IPUM 35050

**FIGURE 10:**
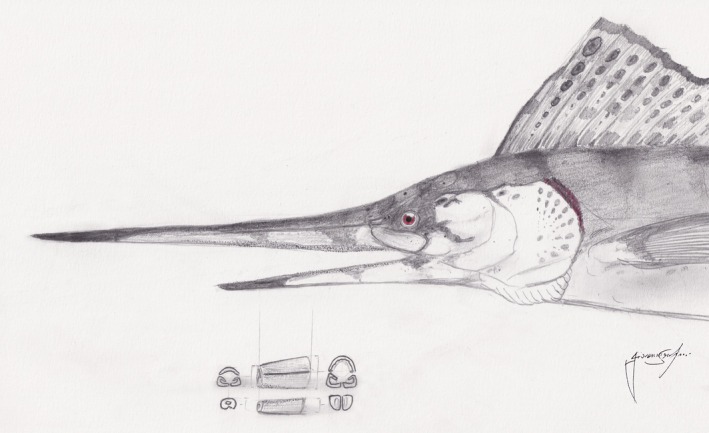
Speculative life restoration of IPUM 35050 (based on *Rhamphoichthys*), with position of the retrieved skeletal remains. Artwork by GS.

Assuming our size estimate to be correct, with the most conservative skull size length calculation of at least 70 cm, IPUM 35050 currently represents one of the largest plethodid specimens ever described. Whereas the morphologically similar and possibly closely related *Rhamphoichthys* is described based on specimens with a total body length of about 1 m (WMNM P 64279 80 cm body length but missing about 15–20 cm rostrum as seen in the referred specimen WMNM P 48342; El Hossny et al., 2023), IPUM 35050 must have had a total body length exceeding 2.5 meters. Plethodids are notoriously large fishes, with some species of *Pentanogmius* in the 170–200 cm body length range (*P. evolutus* with 200 cm estimated total body length (TL); Taverne, 2000b; *P. fritschi* with 175 cm (TL); Shimada, 2016) and with some estimates for other members of the family at approximately 3 meters (Taverne & Gayet, 2005). Among longirostrine plethodids, IPUM 35050 stands out as the largest specimen/taxon, as *Martinichthys* spp. are also described from smaller specimens, with rostra not much longer than 9 cm (Taverne 2000a; El Hossny et al., 2023). We note that some Cenomanian specimens previously attributed to “*Protosphyraena” stebbingi* and “*P.” minor* are represented by terminal rostral portions in the range of 40–45 cm length (Woodward, 1909; Amalfitano et al., 2019; El Hossny et al., 2023 supplementary), more consistent with the large sizes of IPUM 35050. However, the taxonomic position of these specimens is problematic, and we prefer to wait for further dedicated studies before including them in a plethodid body size frame. With its large size, long and deep rostrum, light‐cancellous bone histology and possible presence of an oil gland to reduce drag, IPUM 35050 likely represented a fast‐swimming pelagic predator of smaller (possibly schooling) fishes, with body proportions and an ecological role similar to those of modern sailfishes and marlins (Figure 10). The outstanding convergence of rostral and dental characters among the two taxa supports this interpretation. This ecomorphotype is also supported by the paleoenvironmental context, the open Ligurian–Piedmont ocean, a deep oceanic setting far from the mainland where this taxon would have hunted with fast cruising in the epipelagic zone. A strictly pelagic (or even exclusively oceanic) distribution of this taxon would also explain the rarity of its remains in the Cretaceous marine fossil record of vertebrates, as late Mesozoic deep‐water settings are less common and less studied than their neritic counterparts (Serafini et al., 2024). Moreover, the taphonomy of low‐sedimentation rate settings usually prevents fossilization processes (Allison et al., 1991; Serafini et al., 2024), resulting in the very rare occurrence of specimens such as IPUM 35050. The occurrence of a highly derived and open‐ocean specialized plethodid tselfatiiform in upper Campanian‐lower Maastrichtian strata corroborates the survival of the group beyond the end of the Campanian, previously only highlighted by the description of a single Maastrichtian plethodid specimen from Morocco by Cooper and Norton (2023). It is interesting to note that a similar ecomorphotype of IPUM 35050 in the Late Cretaceous is also shared by the hyposcormine pachycormid *Protosphyraena* spp., with species being reported also in Campanian‐Maastrichtian strata (Friedman, 2012; Kanarkina et al., 2025). As both *Protosphyraena* species and longirostrine tselfatiiforms coexisted in marine ecosystems at least since the Cenomanian (occurrence of *Rhamphoichthys*), the two similar ecotypes were probably specialized for different ecological niches to avoid competition, like modern sailfishes, marlins and swordfishes (Domenici et al., 2014; Habegger et al., 2020).

## 6 CONCLUSIONS

In this study we report the occurrence of a large, undetermined taxon of plethodid tselfatiiform from the middle upper Campanian–lower Maastrichtian of Northern Italy. The specimen shares rostral features with Cenomanian longirostrine plethodids like *Rhamphoichthys* and *Martinichthys*, including the presence of an elongated upper jaw composed of premaxillae and mesethmoid, but differs from other plethodids by the following unique features:

- pronounced reticular ornamentation of jaw bones,
- presence of a large triangular vacuity in the upper jaw,
- distinctive dental morphotypes based on presence and pronunciation of apicobasal ridges, possibly linked to different erupting stages,
- distinctive presence of acrodin capping on the teeth (tough the feature might be common in Plethodidae but had been simply overlooked up to now),
- presence of a fossa at the premaxilla‐mesethmoid suture associated with an inner longitudinal canal.

By comparison with internal osteological correlates in the rostrum of extant oil gland‐bearing xiphioids, we hypothesize that the studied specimen could have hosted a similar glandular structure at the base‐midline of its upper jaw. As a collateral result of our comparative survey of these structures in extant xiphioids, we also report the occurrence of large glandular sinuses already in postlarval specimens of *X. gladius* and *I. platypterus*.

Additional craniodental convergent traits shared by the plethodid described herein and modern billfishes are represented by the following:

- rostral proportions,
- similar dental morphology and attachment modes,
- consistent histological mesostructure of premaxilla and dentary walls, likely associated with fat stroma.

The studied specimen, possibly representing a new taxon, is therefore reconstructed as a large, fast‐swimming, longirostrine pelagic predator with an ecomorphology extremely similar to that of Cenozoic and Recent billfishes. The present study strongly corroborates how some derived members of Tselfatiiformes independently acquired a billfish‐like morphology, suggesting that a pelagic environment and piscivorous diet based on schooling fishes are capable of driving the development of similar structures and body plans in distantly related teleost lineages.

## AUTHOR CONTRIBUTIONS

**Conceptualization:** Giovanni Serafini. **Data curation:** Giovanni Serafini. **Formal analysis:** Giovanni Serafini, Jürgen Kriwet, Tommaso Toldo, and Eliana Fornaciari. **Funding acquisition:** Giovanni Serafini. **Investigation:** Giovanni Serafini, Jürgen Kriwet, Jacopo Amalfitano, Tommaso Toldo, Giorgio Carnevale, and Eliana Fornaciari. **Methodology:** Giovanni Serafini, Eliana Fornaciari, Giorgio Carnevale, Jürgen Kriwet, and Tommaso Toldo. **Supervision:** Giorgio Carnevale and Jürgen Kriwet. **Visualization:** Giovanni Serafini. **Writing—original draft preparation:** Giovanni Serafini. **Writing—review and editing:** Giovanni Serafini, Jürgen Kriwet, Jacopo Amalfitano, Giorgio Carnevale, and Eliana Fornaciari.

## ETHICS STATEMENT

No ethical requirements for research animals in this study were needed, as the main surveyed specimen consists of fossilized material. Data from comparative extant animals were sourced from digitalized wet specimens and from osteological collections; no animal was specifically sacrificed for this study.

## Supporting information


**Supplementary S1:** TXRF spectra of IPUM 35050 skeletal tissue.
**Supplementary S2:** Isolated plethodid tooth (VP‐15224, Sternberg Museum) from the Upper Cretaceous of the Greenhorn‐Lincoln formation (Las Animas, Colorado, US). Courtesy of Tamara El Hossny.

## Data Availability

The data that support the findings of this study are available from the corresponding author upon reasonable request.
