# Supplementary material for: Almost billfish: convergent longirostry, micro‐dentition, and possible glandular sinuses in a large teleost fish from the Upper Cretaceous of Northern Italy
Source: J Anat. 2025 Jun 25;247(6):1109–30. doi: 10.1111/joa.14290 (PMC12588171; doi:10.1111/joa.14290)
Supplement: Supplementary file 1 — Supplementary S1: TXRF spectra of IPUM 35050 skeletal tissue. Supplementary S2: Isolated plethodid tooth (VP‐15224, Sternberg Museum) from the Upper Cretaceous of the Greenhorn‐Lincoln formation (Las Animas, Colorado, US). Courtesy of Tamara El Hossny. [file JOA-247-1109-s001.pdf]

"Almost billfish: convergent longirostry, micro-dentition, and possible glandular sinuses in a large teleost fish from the Upper Cretaceous of Northern Italy"

Giovanni Serafini, Jürgen Kriwet, Tommaso Toldo, Eliana Fornaciari, Jacopo Amalfitano, Giorgio Carnevale

Supplementary material

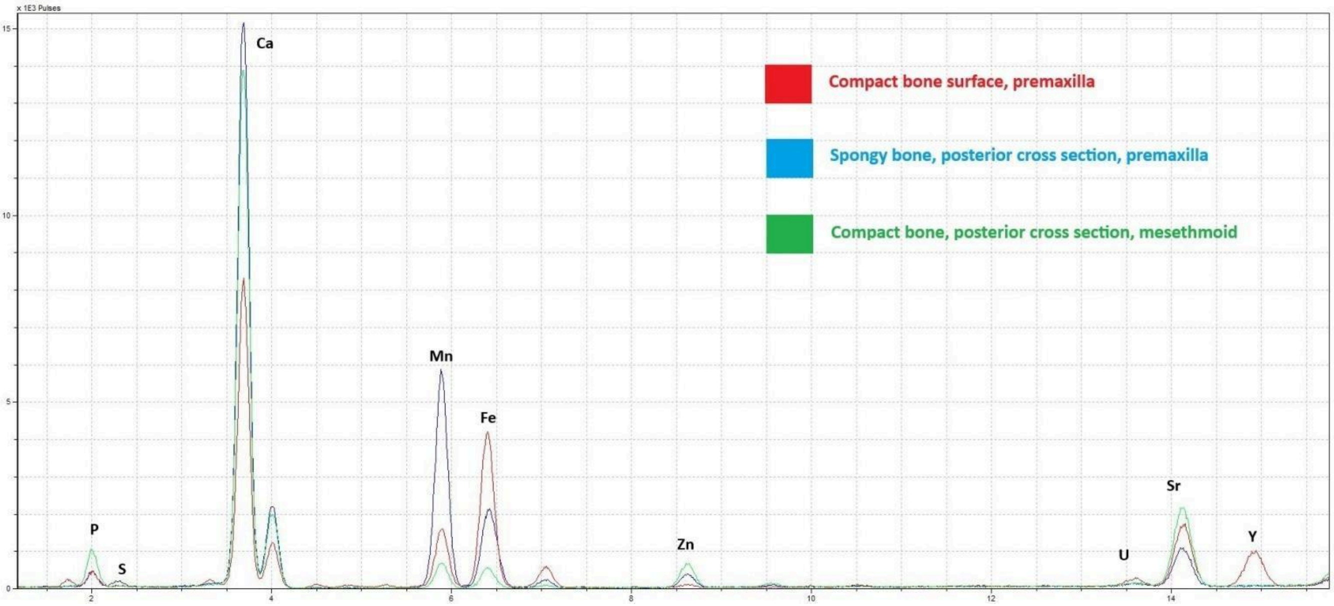

Supplementary S1: TXRF spectra of IPUM 35050 skeletal tissue.

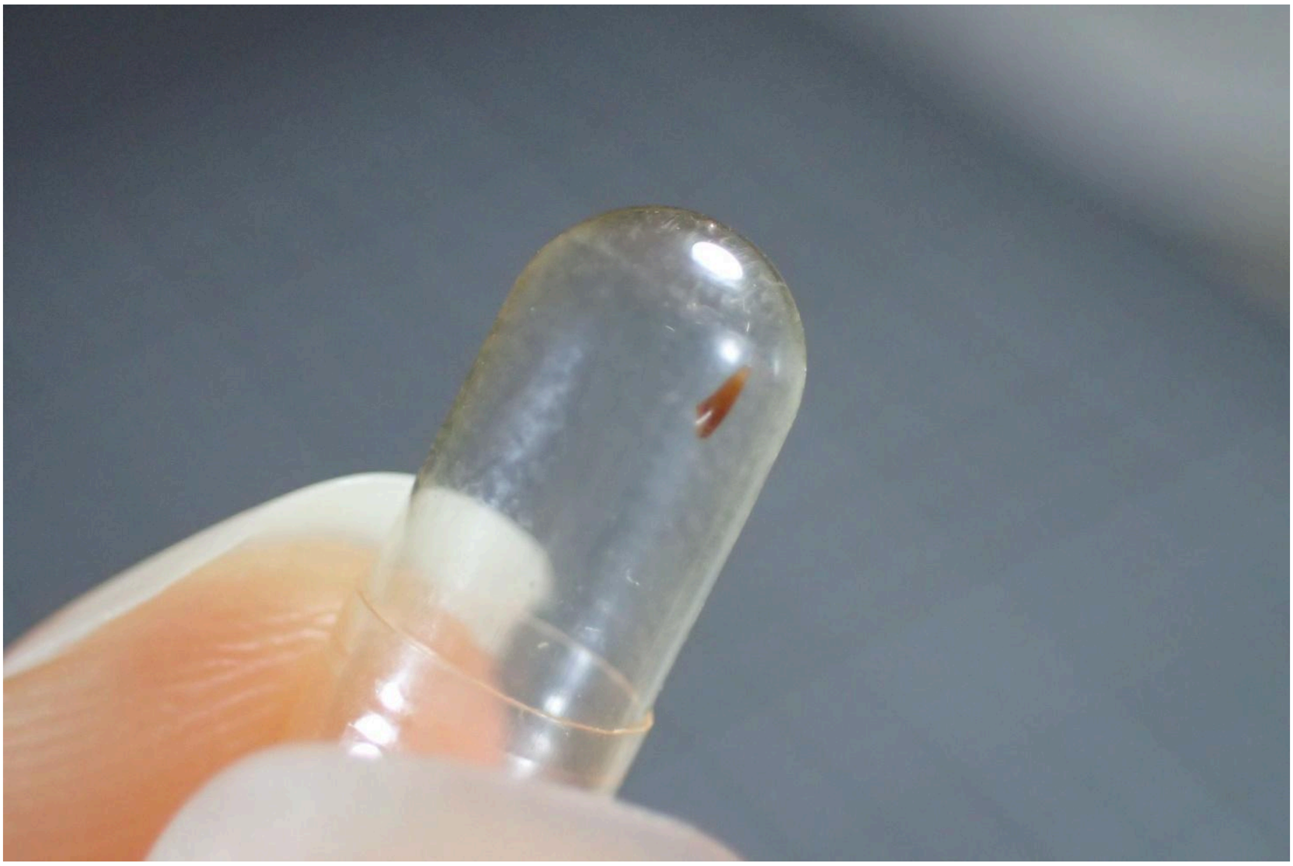

Supplementary S2: Isolated plethodid tooth (VP-15224, Sternberg Museum) from the Upper Cretaceous of the Greenhorn-Lincoln formation (Las Animas, Colorado, US). Courtesy of Tamara El Hossny.
